# Proteostasis in the Endoplasmic Reticulum: Road to Cure

**DOI:** 10.3390/cancers11111793

**Published:** 2019-11-14

**Authors:** Su Min Nam, Young Joo Jeon

**Affiliations:** 1 Department of Biochemistry, Chungnam National University College of Medicine, Daejeon 35015, Korea; sumin4916@hanmail.net; 2 Department of Medical Science, Chungnam National University College of Medicine, Daejeon 35015, Korea

**Keywords:** endoplasmic reticulum (ER) stress, unfolded protein response (UPR) of the ER, ER-associated protein degradation (ERAD), protein quality control, proteostasis, cancer, therapeutic targets

## Abstract

The endoplasmic reticulum (ER) is an interconnected organelle that is responsible for the biosynthesis, folding, maturation, stabilization, and trafficking of transmembrane and secretory proteins. Therefore, cells evolve protein quality-control equipment of the ER to ensure protein homeostasis, also termed proteostasis. However, disruption in the folding capacity of the ER caused by a large variety of pathophysiological insults leads to the accumulation of unfolded or misfolded proteins in this organelle, known as ER stress. Upon ER stress, unfolded protein response (UPR) of the ER is activated, integrates ER stress signals, and transduces the integrated signals to relive ER stress, thereby leading to the re-establishment of proteostasis. Intriguingly, severe and persistent ER stress and the subsequently sustained unfolded protein response (UPR) are closely associated with tumor development, angiogenesis, aggressiveness, immunosuppression, and therapeutic response of cancer. Additionally, the UPR interconnects various processes in and around the tumor microenvironment. Therefore, it has begun to be delineated that pharmacologically and genetically manipulating strategies directed to target the UPR of the ER might exhibit positive clinical outcome in cancer. In the present review, we summarize recent advances in our understanding of the UPR of the ER and the UPR of the ER–mitochondria interconnection. We also highlight new insights into how the UPR of the ER in response to pathophysiological perturbations is implicated in the pathogenesis of cancer. We provide the concept to target the UPR of the ER, eventually discussing the potential of therapeutic interventions for targeting the UPR of the ER for cancer treatment.

## 1. Introduction

**Figure 1:**
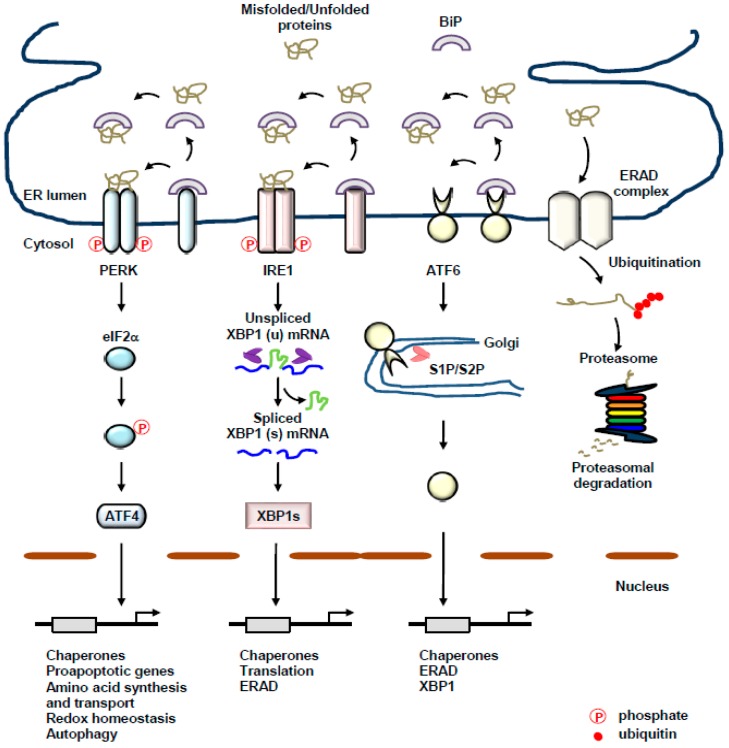
The unfolded protein response (UPR) of the endoplasmic reticulum (ER) and ER-associated degradation (ERAD). The UPR of the ER is an adaptive interplay of signal transduction pathways to coordinate ER stress response and to relieve ER stress, resulting in the re-establishment of proteostasis. The UPR consists of three stress sensors localized at the ER membrane, activating transcription factor 6 (ATF6), inositol-requiring protein 1 (IRE1), and protein kinase RNA (PKR)-like ER kinase (PERK). Under normal conditions, these stress sensors are maintained in an inactive form via the direct binding of a chaperone, binding immunoglobulin protein (BiP) to the luminal domain of the stress sensors. ER stress-induced release of BiP from the stress sensors leads to the activation of the UPR. ERAD is conserved protein degradation machinery of the ER to remove unfolded, misfolded, or unassembled proteins by the cytosolic ubiquitin-proteasome system (UPS).

The endoplasmic reticulum (ER) is a specialized organelle composed of flattened discs and elongated tubules. The ER is not only involved in biosynthetic processes, but also coordinates signal-sensing, -integrating, and -transducing processes to maintain cellular homeostasis. The ER orchestrates the synthesis, folding, maturation, and stabilization of proteins embedded in the plasma membrane or destined to be secreted, which constitute around one-third of total proteins that are synthesized in the cell [1,2]. Additionally, the ER is involved in a variety of cellular processes, including the maintenance of Ca^2+^ homeostasis, detoxification, the biosynthesis of lipid species, and the degradation of glycogen [3,4,5], indicating the involvement of the ER in signal transduction, lipid metabolism, and cell–cell communications. A large variety of physiological and pathological perturbations, including an increase in protein synthesis, impaired ubiquitin-proteasome system (UPS), defects in autophagy, ER-Ca^2+^ depletion, hypoglycemia, energy deprivation, dysregulated redox homeostasis, inflammatory stimuli, and hypoxia may interfere with ER homeostasis, thereby leading to the accumulation of misfolded proteins in the ER, which is referred to as ER stress. In response to ER stress, the ER activates unfolded protein response (UPR) of the ER to integrate ER stress signals (Figure 1) [6]. The activated UPR organizes the temporal decrease in protein synthesis in the company with a subset of gene expression that is involved not only in the folding, maturation, and stabilization of proteins, but also in protein degradation via ER-associated degradation (ERAD) to re-establish protein homeostasis, also termed proteostasis. Intriguingly, deregulation of the UPR and a subsequent failure in the re-establishment of proteostasis are closely linked with a various human diseases, including cardiovascular diseases, neurodegenerative diseases, immune diseases, and cancer [7,8,9], implicating the essential role of the UPR as a stringent protein quality-control machinery of the ER. Even with the assistance of the dedicated UPR, if ER stress is severe and not resolved, the UPR activation switches from an adaptive pro-survival to a toxic pro-death response [10].

In this review, we not only highlight new insights into how protein quality control of the ER to pathophysiological perturbations is implicated in the pathogenesis of cancer, involving tumor development, angiogenesis, aggressiveness, immunosuppression, and therapeutic response of cancer, but also discuss the current state of therapeutic interventions for targeting UPR of the ER in cancer.

## 2. The UPR of the ER: A Complex Interplay between Three Transmembrane ER-Resident Stress Sensors

The UPR of the ER is an elaborate interplay of signal transduction pathways, which senses ER stress and transduces the ER stress signals from the ER to the nucleus and cytoplasm, thereby coordinating ER stress response and restoring the capability of the ER to adequately fold or eventually eliminate misfolded, unfolded, or unassembled proteins, which re-establishes ER homeostasis. The UPR is composed of three major stress sensors localized at the ER membrane, including activating transcription factor 6 (ATF6) α and β, inositol-requiring protein 1 (IRE1) α and β, and protein kinase RNA (PKR)-like ER kinase (PERK) [11,12,13]. The expression of IRE1α is found in almost all of tissues, whereas the expression of IRE1β is restricted to the epithelial cells of gastrointestine [14]. IRE1 and PERK belonging to type I transmembrane proteins have a cytosolic Ser/Thr kinase domain and an ER luminal domain, while ATF6 belonging to a type II transmembrane protein possess an ER luminal domain and a cytosolic cyclic AMP response element-binding protein (CREB)-ATF basic leucine zipper domain [9]. Under normal condition, these three stress sensors are sequestered in an inactive form via the direct interaction with a chaperone belonging to a heat shock protein 70 family, binding immunoglobulin protein (BiP, also known as GRP78) [15]. Under the condition in which the accumulation of unfolded or misfolded proteins are above a threshold of the folding capacity of the ER, known as ER stress, BiP is dissociated from the ER stress sensors and is recruited to misfolded or unfolded proteins, resulting in the priming of the stress sensors for activation [16,17]. The UPR sensors can also be regulated by protein disulfide isomerases (PDIs) [18,19,20], suggesting the existence of a sophisticated interplay for the activation of the UPR. Furthermore, direct interaction of misfolded or unfolded proteins with PERK or IRE1 has been suggested to activate PERK or IRE1 [11,21,22,23]. 

### 2.1. PERK

PERK is a type I transmembrane Ser/Thr kinase with a luminal stress-sensing domain and a cytosolic kinase domain [24]. ER stress-induced release of PERK from BiP leads to the homodimerization and trans-autophosphorylation of PERK, which activates the kinase domain of PERK. Eukaryotic translation initiation factor 2 α (eIF2α) and nuclear factor-E2-related factor 2 (Nrf2) are known to be substrates for PERK [25]. The activated PERK phosphorylates the eIF2α at serine 51, which leads to the inhibition of cap-dependent translation and the reduction in global protein translation, thereby decreasing the amount of newly synthesized proteins inside the ER, which ensures the cell will relieve the ER stress. In contrast, cap-independent translation can be facilitated by PERK-mediated eIF2α phosphorylation [24,25]. Activation transcription factor 4 (ATF4, also known as CREB2) is favorably translated under the condition of ER stress, thereby leading to the transactivation of various genes, including growth arrest and DNA damage-inducible protein (*Gadd34*), ER oxidoreductin 1 (*Ero1*) and CCAAT/enhancer-binding protein (C/EBP) homologous protein (*Chop*), all of which not only fine-tune the redox and metabolic status of the ER, subsequently providing a suitable oxidative environment of the ER for proper protein folding, but also promote autophagy and apoptosis [25,26,27].

PERK-mediated phosphorylation of Nrf2 in response to ER stress promotes the upregulation of a number of genes involved in redox homeostasis by stimulating the release of Nrf2 from its repressor, kelch-like enoyl-CoA hydratase (ECH)-associated protein 1 (KEAP1), in the cytoplasm and the subsequent translocation of Nrf2 into the nucleus [28]. Therefore, these parallel PERK-eIF2α-ATF4 and PERK-Nrf2 pathways may resolve ER stress, restore the folding capacity of the, and facilitate adaptation to oxidative stress.

### 2.2. IRE1

IRE1 is a type I transmembrane kinase and have both of an endoribonuclease activity and a Ser/Thr kinase activity within its cytoplasmic domain, although IRE1 itself is the only known direct substrate phosphorylated by IRE1 [29]. ER stress-mediated release of IRE1 from BiP facilitates the oligomerization and trans-autophosphorylation of IRE1, thereby leading to a conformational change and subsequent activation of endonuclease domain of IRE1. Additionally, an ER chaperone, heat shock protein 47 (HSP47), binds to the luminal domain of IRE1 and blocks the interaction between IRE1 and BiP, subsequently promoting the activation of IRE1 [30,31]. IRE1 non-conventionally splices unspliced X-box binding protein 1 (*XBP1*) mRNA (*Xbp1u*) and, therefore, a translational frameshift is formed to generate spliced *XBP1* mRNA (*Xbp1s*) [22,32]. As a potent transcription factor, XBP1s facilitates the expression of a variety of genes involved in ER protein quality control, ERAD, ER/Golgi biogenesis, redox homeostasis, and oxidative stress response [33,34].

### 2.3. ATF6

ATF6 is a type II transmembrane protein with a cytosolic bZIP transcription factor domain. Upon ER stress, the dissociation of ATF6 from BiP results in its translocation to the Golgi apparatus, in which ATF6 is cleaved by the Golgi enzymes site 1 protease (S1P) and S2P. This processing of ATF6 produces a cleaved cytosolic p50 fragment and as an active transcription factor, the cytosolic p50 fragment upregulates the expression of XBP1 and the genes implicated in protein folding and ERAD processes, thereby leading to the improvement of the folding capacity of the ER, the elimination of the unfolded or misfolded proteins, and the subsequent restoration of proteostasis [35,36]. Furthermore, cytosolic p50 fragment of ATF6 is also responsible for ER expansion as well as lipid biogenesis [37,38].

## 3. ER-Mitochondria Interconnection and UPR

The ER is interconnected with almost all of other cellular organelles and operates with these organelles to sense extrinsic and intrinsic perturbations, integrate the stress signals, and finetunes cellular signal transduction processes, indicating that the ER is a central coordinator to ensure cellular homeostasis [39]. Specifically, tight interconnection between the ER and mitochondria plays a multifaceted roles in the regulation of fundamental physiological processes, involving cell fate decisions, mitochondrial bioenergetics, proteostasis, and metabolism, which is closely associated with tumorigenesis and therapeutic responses of cancer cells. The crosstalk between the ER and mitochondria is tightly controlled by microdomains referred to as mitochondria-associated ER membranes (MAMs) [40,41,42,43]. Intriguingly, MAMs are not only static physical bridges between the ER and mitochondria, but also essential platforms for the exchange of molecular signals and the formation of protein complex for critical decisions in response to perturbations of cellular homeostasis. Further, over the past years, it has been demonstrated that oncogenes as well as tumor suppressors are localized in MAMs and exert pro- and anti-apoptotic functions via the regulation of the transfer of Ca^2+^ and the communications between the ER and mitochondria.

Accumulating evidence demonstrates that MAMs play a pivotal role not only in the control of ER stress, but also in the intense and mutual crosstalk between the UPR of the ER and the complex signaling processes of mitochondria [44,45,46]. It has been demonstrated that a variety of ER chaperones, involving BiP, calnexin, calreticulin, and sigma 1 receptor (Sig1R) are localized in MAMs [47,48]. PERK has been shown to be an integral member of MAMs and PERK depletion has been known to result in the weakness of ER-mitochondria contact sites, thereby leading to the increased resistance to apoptosis upon ER stress [49]. Further, PERK-ATF4 axis is required for the induction of a truncated variant of sarco/endoplasmic reticulum Ca^2+^ ATPase 1 (SERCA1), S1T, that is localized to MAMs, increases the number of ER-mitochondria contact sites and mitochondrial Ca^2+^ overload, and attenuates mitochondrial movement, which consequently promotes apoptosis, suggesting that PERK-ATF4 axis reinforces MAMs [50]. Additionally, PERK-ATF4 axis upregulates the expression of E3 ubiquitin ligase Parkin that is reported to increase ER-mitochondria interconnection, suggesting the key role of the PERK-ATF4 axis in the upregulation of MAMs-resident proteins [51]. 

IRE1 has been also shown to be localized in MAMs. Upon ER stress, the association of IRE1 with the MAMs-resident ER chaperone Sig1R promotes IRE1 dimerization [48]. Additionally, Sig1R and BiP form a Ca^2+^-sensitive complex and prolong Ca^2+^ signaling by stabilizing inositol-1,4,5-triphosphate receptor (IP_3_R) [48].

Although the relationship between MAMs and tumorigenesis remains to be elucidated, given that cancer cells are addicted to ER-mitochondria interconnections and ER-mitochondrial Ca^2+^ transfer, targeting of MAMs structure, functions, and dynamics represents potential therapeutic strategy for the treatment of cancer.

## 4. Cell Fate Decisions and UPR

**Figure 2:**
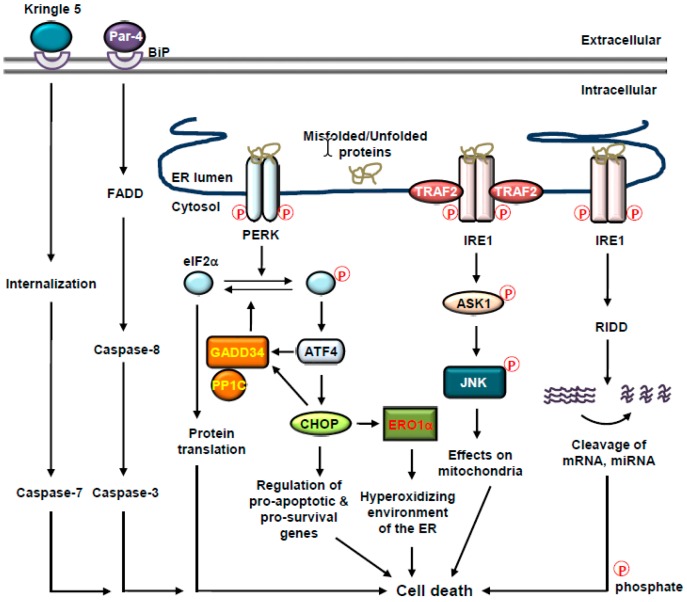
The unfolded protein response (UPR) and its connection to cell death. Under severe endoplasmic reticulum (ER) stress, sustained protein kinase RNA (PKR)-like ER kinase (PERK) activation is required for the transition from protective to pro-apoptotic UPR function. Cell-surface binding immunoglobulin protein (BiP) forms a complex with Kringle 5, enhancing caspase-7-mediated cell death. In addition, extracellular prostate apoptosis response-4 (Par-4) binds to cell-surface BiP, thereby leading to apoptosis via activation of Fas-associated protein with death domain (FADD)/caspase-8/caspase-3 pathway. Upregulated CCAAT/enhancer-binding protein (C/EBP) homologous protein (CHOP) regulates the expression of pro-apoptotic and pro-survival genes, thereby leading to cell death. CHOP also mediates cell death via the upregulation of the expression of ER oxidoreductin 1 (ERO1α) and growth arrest and DNA damage-inducible protein (GADD34). As a molecular scaffold, inositol-requiring protein 1 (IRE1) is responsible for the recruitment of an E3 ubiquitin ligase, tumor necrosis factor (TNF) receptor-associated receptor 2 (TRAF2), and for the activation of mitogen-activated protein kinase (MAPK) signaling pathways, triggering cell death. In addition, regulated IRE1-dependent decay (RIDD)-mediated cleavage of miRNAs and mRNAs induces cell death.

The UPR of the ER is connected to cell fate decisions. Under tolerable ER stress, UPR activation facilitates cell survival through the relief of ER stress and the restoration of homeostasis. However, when the adaptive responses of the UPR is overwhelmed by severe and persistent ER stress and ER homeostasis is not restored, the responses of the UPR change over from adaptive pro-survival to toxic pro-death and/or premature senescence as two tier safety mechanisms via the release of Ca^2+^, the upregulation of pro-apoptotic B cell chronic lymphocytic leukemia (CLL)/lymphoma 2 (BCL-2) family members, the production of reactive oxygen species (ROS), or the regulation of microRNAs [11,52]. Although the exact switching mechanisms remain largely elusive and has begun to be understood, potential mechanisms may be not only based on the modulation of mRNA stability and differential expression of proteins involved in pro-survival and pro-death signals, but also tightly regulated by anti- or pro-apoptotic BCL-2 proteins, which coordinate information about the strength and the duration of ER stress, subsequently transducing the information to adaptive pro-survival or toxic pro-death signaling pathway for cell fate decision [11,52] (Figure 2).

### 4.1. Cell Fate Decisions and BiP

BiP has been demonstrated to be located not only in the lumen of the ER, but also on the surface of tumor cells, raising the possibility that BiP on the tumor cell surface may play a role as a cell surface receptor in signal transduction pathways for cell fate decisions. BiP on the surface of prostate cancer cells activates pro-survival MAPK and Rac-α serine/threonine-protein kinase (AKT, referred to as protein kinase B (PKB)) signaling pathways [53]. On the contrary, a tumor suppressor protein, prostate apoptosis response-4 (Par-4), is secreted from cancer cells and binds to cell surface BiP, subsequently activating extrinsic apoptotic pathway [54]. Furthermore, angiogenesis inhibitor Kringle 5 (K5) interacts with cell surface BiP and promotes apoptosis in tumor cells [55].

### 4.2. Cell Fate Decisions and PERK

Under mild ER stress, transient activation of PERK is involved in pro-survival gene expression. Activated Nrf2 by PERK binds to antioxidant response element (ARE) on the promoter regions of *Bcl-xL* and *BCL-2* and induces the expression of Bcl-xL and BCL-2, thereby leading to the induction of antioxidant defense system and subsequent inhibition of cell death [56,57,58,59]. Additionally, miR-211 induced by ATF4 facilitates histone methylation at the *DDIT3* promoter and subsequently attenuates the expression of CHOP [60].

Under severe ER stress, sustained activation of PERK is responsible for the switch from protective pro-survival to toxic pro-death [61]. CHOP has been suggested to play a crucial role in ER stress-induced cell death under excessive and sustained activation of PERK [27,62]. At early stages of ER stress, CHOP expression is downregulated by Toll-like receptor (TLR) signaling and histone methylation [60,63]. However, if ER stress is prolonged and unresolved, upregulated CHOP increases the synthesis and misfolding of proteins by upregulating the expression of tRNA synthetase, which evokes oxidative stress and subsequent cell death [9,64]. Further, both of the treatment of antioxidant butylated hydroxyanisole and RPL24 depletion not only decreases ROS production, but also protein translation, thereby preventing cell death [64]. Under severe and prolonged ER stress, CHOP-mediated upregulation of ERO1α and GADD34 accelerates cell death. GADD34 forms a feedback loop with protein phosphatase 1C (PP1C) and mediates the dephosphorylation of eIF2α, resulting in the resumption of protein synthesis, which can increase protein load in the ER and therefore amplify toxic pro-death signal [65]. Additionally, CHOP-mediated ERO1α induction creates hyperoxidizing environment of the ER, which is detrimental to adequate protein folding and consequently propagates pro-death signal [27]. ERO1α transfers electrons to molecular oxygen in the course of disulfide bond formation, which generates hydrogen peroxide and subsequently facilitates IP_3_R-mediated Ca^2+^ efflux from the ER and ROS production. Ca^2+^ influx into mitochondria through MAMs and its increase inside mitochondria trigger mitochondrial ROS production, activate nitric oxide synthase and Krebs cycle dehydrogenases, and stimulate the release of cytochrome *c*, suggesting that ER stress attenuates the function of mitochondria and mediates oxidative stress response, potentiating cell death [66]. Additionally, CHOP downregulates the expression of pro-survival BCL-2 and perturbs the cellular redox state, thereby sensitizing cells to apoptosis [67]. CHOP-mediated suppression of BCL-2 results in the release of BCL-2 homology domain 3 (BH3)-only proteins, including BAD, PUMA, and NOXA, resulting in the induction of mitochondria-dependent apoptosis [68,69,70]. Moreover, CHOP-mediated upregulation of BCL-2-interacting mediator of cell death (BIM) induces apoptosis upon ER stress [71]. Additionally, CHOP upregulates death receptor 5 (DR5) and tribbles 3 (TRB3), which sensitizes cells to apoptosis [72,73]. These observations suggest that sustained activation of PERK signaling operates to switch cells from adaptation for survival to cell death.

### 4.3. Cell Fate Decisions and IRE1

IRE1-mediated activation of nuclear factor κ-light-chain-enhancer of activated B cells (NF-κB) and signal transducer and activator of transcription 3 (STAT3) has been shown to upregulate the expression of anti-apoptotic proteins, including myeloid cell leukemia 1 (MCL1) and inhibitor of apoptosis (IAP), the caspase-8 inhibitor cellular FADD-like IL-1β-converting enzyme (FLICE)-inhibitory protein (c-FLIP), and BCL-2 family members, which inhibits cell death [74].

Under tolerable ER stress, IRE1 plays a protective role by non-conventionally splicing *Xbp1u and* generating *Xbp1s.* Interestingly, independent of the non-conventional splicing capability of IRE1, IRE1 is responsible for a molecular scaffold in the formation of UPRosome, in which various adaptor proteins and regulators assemble to modulate the amplitude and kinetics of IRE1 signaling and coordinate the signals for cell fate decisions. UPRosome integrates downstream cellular stress responses, involving protein quality control, ERAD, organelle biogenesis, and autophagy, and eventually decides cell fate [75,76,77,78]. Actually, the amplitude of IRE1 signaling at the ER membrane is modulated by the formation of protein complex composed of BCL-2 family members, regulator and adaptor proteins, and the cytosolic domain of IRE1. IRE1 associates with apoptosis signal regulating kinase 1 (ASK1)-interacting protein 1 (AIP1), resulting in the stimulation of IRE1 signaling [79]. Additionally, ER-resident protein phosphatase 1B (PTP1B) [80] and HSP72 [81] physically interact with IRE1, thereby potentiating IRE1 signaling. As a molecular scaffold, IRE1 is responsible for the recruitment of an E3 ubiquitin ligase, tumor necrosis factor (TNF) receptor-associated receptor 2 (TRAF2), and the activation of its downstream kinase, ASK1, which activates p38 mitogen-activated protein kinase (MAPK) and c-Jun N-terminal kinase (JNK) signaling pathways and subsequently mitochondrial apoptosis [82,83]. C-Jun N-terminal inhibitory kinase (JIK) has been also known to interact with and modulate IRE1-TRAF2 complex [84]. Additionally, IRE1-mediated MAPK activation in turn not only activates pro-apoptotic BH3-only proteins such as BIM, but also attenuates the anti-apoptotic activity of BCL-2 [85]. Furthermore, the direct association of IRE1 with pro-apoptotic BCL-2-associated X protein (BAX) and BCL-2-antagonist/killer (BAK) regulates IRE1 activity and stimulates mitochondrial apoptosis mediated by ER stress [86]. Interestingly, the expression of BAX in BAX and BAK-deficient mouse embryonic fibroblasts (MEFs) could reconstitute IRE1-TRAF2 signaling pathway and BH3-only proteins-facilitated mitochondrial apoptosis [87], suggesting that the crosstalk between BCL-2 protein family members and IRE1 might be a key player of cell fate decisions upon ER stress.

Prolonged ER stress has been shown to inactivate UPRosome signaling pathway. IRE1 signaling pathway is downregulated via a direct interaction between the cytosolic region of IRE1 and BAX inhibitor 1, BI-1, in different settings [88]. For example, BI-1 displaces BAX and BAK from the UPRosome or alternatively interacts with BAX and BAK and subsequently inhibits the association of BAX and BAK with IRE1, resulting in the inactivation of UPRosome signaling [88,89,90,91]. Interestingly, it has been shown that ER-associated E3 ubiquitin ligase, bifunctional apoptosis regulator (BAR), associates with BI-1, which leads to the proteasomal degradation of BI-1 and the sustained activation of IRE1 signaling [92].

Under non-resolvable ER stress, a large variety of ER-bound mRNAs involving *IRE1* can be degraded by regulated IRE1-dependent decay (RIDD). RIDD is a well-conserved mechanism, in which IRE1 cleaves mRNA transcripts possessing the consensus sequence, CUGCAG, in the company of a stem-loop structure [93,94,95,96]. In addition, under severe and prolonged ER stress, IRE1 can be hyperactivated and cleave microRNAs such as miR-17, miR-34a, miR-96, and miR125b that normally repress pro-apoptotic targets including pro-apoptotic caspase-2, which activates caspase-2 and induces caspase-2-mediated cleavage of BH3 interacting-domain death agonist (BID), thereby facilitating BAX and BAK-dependent apoptosis [97,98,99].

### 4.4. Cell Fate Decisions and ER-Associated Caspases

Several ER-related caspases have been suggested to be implicated in ER stress-induced apoptosis [84,100]. Although caspase-12 has been shown to be involved in ER stress-induced apoptosis in rodents, it is not likely that this mechanism operates in humans [100,101,102,103]. ER membrane-localized human caspase-4 is cleaved and activated in response to ER stress [104,105]. Cleavage of caspase-4 is not influenced by BCL-2 that inhibits signal transduction pathway of mitochondria, indicating that caspase-4 is dominantly involved in ER stress-promoted apoptosis not in mitochondrial apoptosis [105]. Interestingly, the cleavage of an integral ER membrane protein, B-cell receptor-associated protein 31 (BAP31) by caspase-8, generates a p20 fragment, which facilitates the release of Ca^2+^ from the ER, concomitant accumulation of Ca^2+^ in mitochondria, thereby leading to the recruitment of dynamin-related protein 1 (Drp1) into mitochondria [106]. Drp1 recruited into mitochondria promotes mitochondrial fission and mitochondrial apoptosome-mediated apoptosis, suggesting the importance of the crosstalk between the ER and mitochondria for cell fate decisions.

## 5. UPR and Cancer

In the course of tumor development, tumor cells are continuously exposed to a variety of extrinsic and intrinsic perturbations, including an increase in protein synthesis and secretion, deregulated protein degradation, genomic instability, changes in the activation status of tumor suppressors and oncogenes, nutrient deprivation, hypoxia, and acidosis, all of which induce ER stress and subsequently activate the UPR. [107,108,109]. The activated UPR has been demonstrated to be closely linked to tumor development, remodeling of tumor microenvironment, angiogenesis, aggressiveness, immunosuppression, and therapeutic response of cancer [109,110]. Interestingly, the sustained activation of UPR at later stages of tumor development could trigger the tumor to adapt to extrinsic and intrinsic insults and enable the tumor to not only resist to ER stress-mediated apoptosis, but also to survive by facilitating epithelial-to-mesenchymal transition (EMT), metastasis, and angiogenesis, while transient UPR at early stages of tumor development could attenuate tumor progression [110,111,112]. Further, cancer patients with the UPR deregulation have been demonstrated to be associated with poor prognosis, suggesting the potential of the UPR deregulation signature for diagnosis as well as prognosis of cancer patients [113]. However, the UPR in cancer remains to be elusive. To establish the role of the UPR in the course of cancer pathogenesis, it is required to clarify the tumor context-dependent differences in the role of the UPR, the alterations in the expression pattern of UPR components, and the interplay of three arms of the UPR.

### 5.1. UPR and Tumorigenesis

#### 5.1.1. Tumorigenesis and BiP

Cancer cells are often characterized by augmented rates of protein synthesis, resulting in an increase in the expression of chaperones and folding enzymes. Increased expression of BiP has been reported to promote tumorigenesis in various tumors, to regulate therapy resistance, and to be associated with poor outcome and recurrence [114,115,116,117,118,119]. BiP-deficient fibrosarcoma cells show attenuated formation of tumors once xenografted in mice [120]. Interestingly, BiP has been shown to be highly expressed in various tumors due to the ER stress induced by oxygen- and nutrients-deprived tumor microenvironment and to be correlated with tumor growth, invasion, and metastasis, suggesting that ER stress-induced upregulation of BiP in tumors is closely related with the adaptation and the improved tolerance of tumor cells to altered tumor microenvironment [117]. Elevated expression of BiP has been found to be associated with higher pathological grade and aggressive phenotypes of breast cancer [121], indicating that BiP might be used to predict poor prognosis. In addition, circulating antibodies against BiP has been found in sera of prostate cancer patients with aggressive phenotype [122].

On the contrary, upregulation of BiP has been also demonstrated to induce dormancy or senescence. Oncogenic HRAS^G12V^-driven ER stress promotes premature senescence through the increased expression of BiP [123]. Further, BiP expression has been shown to be associated with favorable prognosis in lung cancer and neuroblastoma patients [124,125]. Therefore, it is likely that in early stages of tumorigenesis, upregulation of BiP attenuates tumor progression via senescence or dormancy, while in more advanced stages of tumorigenesis, increased expression of BiP facilitates tumor progression via pro-survival or pro-metastatic signals.

#### 5.1.2. Tumorigenesis and IRE1

IRE1 has been demonstrated to be linked with tumor progression. XBP1s has been reported to be elevated in a variety of tumors, involving breast cancer, hepatocellular carcinoma, lymphoma, and multiple myeloma [126,127,128,129]. XBP1s facilitates tumorigenesis and relapse of tumor in triple negative breast cancer (TNBC) [130]. TNBC cells injected into mice have been demonstrated to develop resistance to chemotherapeutic drugs, doxorubicin and paclitaxel, while XBP1 depletion has been shown to attenuate the resistance and tumor recurrence [130,131]. Proto-oncogene MYC has been shown to interact with XBP1 and potentiate the transcriptional activity of XBP1 in TNBC [132]. Furthermore, MYC also binds to the promoter region of *IRE1* and upregulates the expression of IRE1 and subsequent splicing of *XBP1* [132]. Patient-derived TNBC cells transplanted into mice form fewer tumors when XBP1 was depleted, while patient-derived TNBC cells form more tumors when XBP1 was overexpressed [130], suggesting that XBP1 is important for TNBC tumor initiation and progression. Interestingly, it has been shown that IRE1 not only regulates production, but also secretion of pro-inflammatory cytokines in TNBC cells [133]. Inhibition of IRE1 endonuclease activity attenuates the secretion of pro-inflammatory cytokines and enhances chemotherapeutic drug-mediated tumor suppression, suggesting that inhibition of IRE1 can potentiate the efficacy of chemotherapeutics for TNBC treatment [133]. Further, XBP1 upregulation promotes the expression of nuclear receptor coactivator 3 (NCOA3) and induces resistance of luminal type of breast cancers to anti-hormonal agents [134]. Additionally, elevated expression of XBP1 in multiple myeloma patients is associated with poor survival and clinical outcome [135], suggesting that XBP1 is implicated in tumor progression and response to therapies.

IRE1 has been found to be mutated in some tumors [97,136,137]. Some mutant forms of IRE1 are positively correlated with tumor development, despite their intact endonuclease and kinase activities. Additionally, IRE1 is positively correlated with poor prognosis in pre-B acute lymphoblastic leukemia and glioblastoma [138,139,140,141]. Further, XBP1 forms a transcriptional complex with hypoxia-inducing factor 1α (HIF1α), a key regulator of vascular endothelial growth factor (VEGF), and stimulates angiogenesis in TNBC [130].

#### 5.1.3. Tumorigenesis and PERK

PERK has been shown to be linked with hematological as well as solid tumor development. PERK depletion facilitates tumor development [142,143]. Accelerated protein synthesis and ROS production by PERK trigger cell death, while decreases in protein synthesis and ROS production by RPL24 depletion as well as the treatment of antioxidant inhibit cell death, suggesting the tumor-suppressive role of PERK signaling [64]. In contrast, PERK accelerates tumor progression by stabilizing Nrf2 and regulating redox homeostasis [142,144,145,146,147]. Furthermore, PERK facilitates angiogenesis and tumor development not only by upregulating the expression of VEGF, interleukin-6 (IL-6), fibroblast growth factor 2 (FGF2), platelet-derived growth factor receptor β (PDGFRB), and type I collagen inducible protein (VCIP), which are involved in the generation, growth, and stabilization of vessels, but also by downregulating anti-angiogenic cytokines [143,148].

#### 5.1.4. Tumorigenesis and ATF6

Compared to IRE1 and PERK, ATF6 in cancer is largely unknown. ATF6 has been found to be highly expressed in Hodgkin lymphoma and hepatocellular carcinoma patients [128,149]. Interestingly, ATF6 and eIF2α have been shown to play a pivotal role in the activation of mammalian target of rapamycin complex 2 (mTORC2), subsequently promoting angiogenesis in endothelial cells [150]. Further, ATF6 has been known to be involved in the regulation of cancer cell dormancy. The characteristics of cancer cell dormancy involve cell cycle arrest in the G0/G1 phase, termination of cell division, and entry into quiescence [151]. The reactivation of dormant cancer cells by the resumption of optimal circumstances for cancer cells has been suggested to be a main reason for cancer recurrence after therapies [152]. ATF6 modulates cancer cell dormancy via the activation of Ras homolog enriched in brain (RHEB) and mTOR, in which ATF6 not only plays a role as a key survival factor for quiescent squamous carcinoma cells, but is also pivotal for the adaptation of dormant cells to chemotherapy [153]. Moreover, high expression of ATF6 has been found in recurrent tumors and to be correlated with increased chemoresistance [20,154], suggesting a functional link between ATF6 and cancer cell dormancy and subsequent resistance to treatment.

### 5.2. UPR and Metastasis

It has been shown that BiP depletion attenuates lung metastasis of TNBC cells xenografted in mice, whereas BiP overexpression promotes metastasis [155,156].

IRE1 has been shown to be associated with metastasis. The transcriptional complex of XBP1 with HIF1α elevates the expression of pyruvate dehydrogenase kinase 1 (PDK1) and glucose transporter 1 (GLUT1) that are the downstream genes of HIF1α, which facilitates tumor development and invasiveness of TNBC [130]. In contrast, IRE1 significantly attenuates the expression of proteins related to EMT and invasiveness of glioma, including thrombospondin-1, secreted protein acidic and rich in cysteine (SPARC) and decorin, while IRE1 is positively associated with pro-angiogenic factors such as VEGF-A, IL-1β, IL-6, and IL-8 in malignant glioma [157,158], suggesting that a comprehensive analysis of IRE1 arm of the UPR is pivotal for the adequate elucidation of its role in modulating angiogenesis and invasiveness.

PERK has been demonstrated to be involved in EMT [159]. Moreover, PERK arm of the UPR facilitates the metastasis of breast cancer cells by activating lysosome-associated membrane protein 3 (LAMP3) [160]. Additionally, the upregulation of ATF4 has been shown to modulate matrix metalloproteinases in esophageal squamous carcinoma, promote metastasis, and be closely associated with poor prognosis in cancer patients [161].

### 5.3. UPR and Cancer Immunogenicity

Tumor microenvironment is the environment surrounding tumors and includes signaling molecules, infiltrating immune cells, fibroblasts, endothelial cells, extracellular matrix, and blood vessels. Importantly, the complex interplay of UPR signal transduction pathways in and around the tumor microenvironment has begun to be elucidated and demonstrated to be involved in tumor development and tumor immunosurveillance [159]. Elevated expression of CHOP has been found in tumor-infiltrating myeloid-derived suppressor cells (MDSCs) [162]. CHOP depletion in tumor-infiltrating MDSCs is linked to a decrease in immunosuppression toward T cells. Interestingly, TNF-related apoptosis-inducing ligand receptor (TRAIL-R)-induced cell death is stimulated by CHOP in tumor-infiltrating MDSCs [163], suggesting that PERK-ATF4-CHOP axis is essential for the modulation of cancer immunogenicity. 

Persistent activation of IRE1-XBP1 axis has been demonstrated in ovarian tumor-infiltrating dendritic cells (DCs) [164]. Intriguingly, the ovarian tumor-infiltrating DCs promotes ROS production and subsequently disrupts ER homeostasis, thereby leading to the modulation of cancer immunogenicity. Additionally, XBP1 depletion in tumor-infiltrating DCs confers immunostimulatory and anti-tumoral characteristics on tumor-infiltrating DCs in vivo [165,166,167]. Furthermore, pharmacological inhibition of IRE1 in IL-6 and IL-4-stimulated bone-marrow-derived macrophages downregulates macrophage-mediated cell invasion in vitro [168]. ER stress induced by pharmacological application upregulates the expression of lectin-type oxidized LDL receptor-1 (LOX-1) in neutrophils and confers immunosuppressive characteristics on neutrophils [169,170], suggesting that IRE1 arm of the UPR modulates tumor-associated myeloid cells. However, the role of the UPR in cancer immunogenicity has begun to be elucidated and many key issues remain to be clarified for the improvement of immune-based anti-cancer therapies.

## 6. Targeting the UPR in Cancer

Targeting the UPR has been considered to be a promising therapeutic approach, since the UPR is deregulated in various human tumor types [171]. Therefore, it has begun to emerge to be valuable not only to identify molecules that efficiently modulate three arms of UPR, but also to investigate approaches for therapeutic targeting of three arms of UPR for cancer treatment. Given that in a context-dependent manner, the UPR not only promotes adaptive pro-survival, but also toxic pro-death, identification and development of UPR-targeting compounds that trigger severe ER stress-induced cell death or inhibit the protective cell survival could be potential therapeutic approaches for the treatment of cancers. Additionally, manipulations of ER stress has been shown to possess therapeutic potential in preclinical models of cancer [172]. Intriguingly, therapeutic strategies to target the UPR may synergize the effects of conventional chemotherapies. However, there are conflicting literatures considering the impact of modulating discrete UPR signaling. Inhibition of one arm of the UPR may result in the alteration of the other arms of the UPR. Therefore, there is a need to define UPR signaling networks and the mechanisms that finetune the crosstalk between three arms of UPR in detail for the development of promising compounds to target the UPR in cancer.

### 6.1. Modulation of PERK

PERK has been suggested as a promising therapeutic target for cancer treatment. GSK2606414 is a first-in-class PERK inhibitor that selectively binds to the kinase domain of PERK and traps its kinase domain in its inactive conformation [173]. Interestingly, GSK2606414 has been shown to be orally active and attenuate tumor growth of pancreatic cancer in vivo [173]. Additionally, it has been shown that GSK2656157, an optimized version of GSK2606414, has favorable pharmacokinetics and passes the blood–brain barrier through oral delivery. GSK2656157 inhibits PERK autophosphorylation and modulates amino acid metabolism, vascular perfusion, and blood vessel density, thereby preventing tumor growth in vivo [174]. PERK inhibition also sensitizes hypoxic radioresistant glioblastoma and colon cancer cells in vivo [175], suggesting that UPR targeting may counteract adverse effects of conventional anti-cancer therapies. Further, PERK-mediated activation of Nrf2 has been demonstrated to be involved in the development of multidrug resistance [146]. 

Salubrinal and guanabenz have been demonstrated to target the complex of GADD34 and PP1C and inhibit eIF2α dephosphorylation, thereby leading not only to the activation of caspase and subsequent apoptosis, but also the suppression of cell proliferation and invasion [176,177,178,179].

The integrated stress response inhibitor (ISRIB) is a symmetric bisglycolamide that renders cells resistant to eIF2α phosphorylation, which attenuates the activation of ATF4, although its role in the modulation of tumor progression is yet to be elucidated [180].

### 6.2. Modulation of IRE1

Compounds targeting IRE1 bind to the catalytic core of the endonuclease domain or the ATP-binding pocket of the kinase domain of IRE1. Compounds identified by high-throughput screening for IRE1 endonuclease activity bind to the catalytic core of its endonuclease domain and include salicylaldehyde (3-methoxy-6-bromosalicylaldehyde), MKC-3946, 4µ8C, and STF-083010 [140,141,172,181,182]. Reversible binding of 3-methoxy-6-bromosalicylaldehyde to IRE1 attenuates IRE1-mediated non-conventional splicing of *XBP1u* as well as RIDD in vitro [182]. Further, 3-methoxy-6-bromosalicylaldehyde attenuates tunicamycin-induced *XBP1* mRNA splicing in the kidney, liver, and spleen in vivo [182]. It has been demonstrated that MKC-3946 combined with the proteasome inhibitor bortezomib synergistically inhibits the tumor formation of multiple myeloma in vivo, suggesting that MKC-3946-inhibited splicing of *XBP1u* potentiates the ER stress induced by bortezomib [140]. The binding of 4µ8C to lysine 907 residue in the catalytic core of the endonuclease domain leads to the formation of a stable imine, which attenuates IRE1-mediated splicing of *XBP1u* and RIDD [141,181]. STF-083010 has been shown to attenuate the growth of multiple myeloma xenografted in mice [183]. Interestingly, STF-083010 significantly decreases the resistance of breast cancer to tamoxifen in combination with tamoxifen [184]. N^9^-(3-(dimethylamino) propyl)-N^3^,N^3^,N^6^,N^6^-tetramethylacridine-3,6,9-triamine (3,6-DMAD) blocks IRE1 oligomerization as well as its RNase activity, subsequently leading to cytotoxicity in multiple myeloma cell lines [185]. Additionally, B-I09 has been shown to modulate the aggressiveness of chronic lymphocytic leukemia cells in vivo [170].

A class of molecules, referred to as hydroxy-aryl-aldehydes (HAA) has been shown to selectively inhibit IRE1 RNase activity, suggesting the potential of HAA for cancer treatment [186].

Toyocamycin, produced by an *Actinomycete* strain, has been identified as a potent inhibitor of IRE1 RNase activity by using an XBP1 luciferase activity assay [187]. Similar to MKC-3946, toyocamycin shows synergistic effects with bortezomib not only on apoptosis of multiple myeloma cells, but also on the retarded tumor growth of multiple myeloma in vivo [187].

Compounds that bind to the ATP-binding pocket within the kinase domain of IRE1 and inhibit its kinase activity include sunitinib, APY29, quercetin, and compound 3 [97,188]. As type I IRE1 kinase inhibitors, sunitinib and APY29 stabilize ATP-binding pocket of IRE1 as in an active conformation, whereas as type II kinase inhibitors, compound 3 and quercetin stabilize IRE1 as in an inactive conformation by competing with ATP for the binding to IRE1, which inhibits the oligomerization, endonuclease activity, and kinase activity of IRE1 [189]. However, there is no evidence that these inhibitors have a potential as anti-cancer drugs, despite their inhibitory effects on IRE1.

Resveratrol, a natural phenol found in multiple berries, has been shown to reduce the DNA-binding capacity of XBP1, thereby promoting the death of multiple myeloma cells and hepatocellular carcinoma models [190,191].

It has been indicated that XBP1s can be regulated by several posttranslational modifications, involving phosphorylation, acetylation, ubiquitination, and SUMOylation [192], suggesting that targeting of the posttranslational modifications could be potent pharmacological approaches to modulate XBP1.

### 6.3. Modulation of ATF6

ATF6 has been shown to be an important survival factor in dormant squamous carcinoma cells [153]. ATF6 induces the expression of RHEB, which activates mTOR signaling and renders therapeutic resistance to dormant cancer cells, suggesting targeting ATF6 might be one of the valuable therapeutic strategies. 

Ceapins belonging to pyrazole amides have been demonstrated to specifically inhibit the ATF6 by blocking ATF6 processing and its nuclear translocation [193].

### 6.4. Modulation of ERAD

ERAD is the sophisticated protein degradation machinery of the ER to eliminate unfolded, misfolded, unassembled, or tightly regulated proteins by the cytosolic UPS [115,194,195,196]. Targeting ERAD has been demonstrated to induce severe ER stress, to inhibit cell survival, and to stimulate cell death in tumors, suggesting the inhibitors of ERAD might be used as valuable anti-cancer drugs. The first Food and Drug Administration (FDA)-approved proteasome inhibitor, bortezomib, is known to trigger ER stress and is used as an anti-cancer drug for the treatment of lymphoma and multiple myeloma. Bortezomib directly inhibits the proteasome and facilitates cell death [197,198]. Additionally, the cytotoxic effects of bortezomib have been confirmed in different types of malignant cells, including lung, breast, prostate, and colon cancer [199,200]. Further, bortezomib induces the activation of the UPR and cell death by promoting pro-apoptotic ROS signaling pathways [201]. Interestingly, bortezomib has been demonstrated to potentiate not only doxorubicin-induced cell death in hepatoma and large B cell lymphoma in mice, but also the anti-cancer effect of cisplatin via JNK-dependent mechanism, indicating that bortezomib improves the efficacy of chemotherapeutic agents [199,202,203]. Additionally, bortezomib attenuates the secretion of IL-6 and VEGF by endothelial cells [204] and decreases vessel density in xenografts of squamous cell carcinoma [205], suggesting that bortezomib may target tumor-associated angiogenesis. Bortezomib treatment has been shown to reduce microvessel density in six of nine patients with multiple myeloma, which is positively correlated with a better prognosis [206], suggesting that bortezomib negatively modulates angiogenesis and the anti-angiogenic activity could be used as a prognostic marker for the evaluation of therapeutic effectiveness of bortezomib.

Given that toxicities and drug resistance have been demonstrated in bortezomib-treated patients, despite the clinical success of bortezomib [207], a second generation of proteasome inhibitors were designed and generated. BU-32 was shown to have cytotoxic efficacy in multiple myeloma as well as breast cancer cells [208,209]. Further, carfilzomib, marizomib, MLN9708, and salinosporamide have also been developed as proteasome inhibitors and are under clinical trials for the treatment of chronic lymphocytic lymphoma, myeloma bone disease, and multiple myeloma [200,210,211,212,213,214,215]. Interestingly, carfilzomib was applied in combination with carboplatin and etoposide in a clinical trial for relapsed small-cell lung cancer [216].

The inhibition of proteasome by the protease activity of nelfinavir itself leads to the accumulation of polyubiquitinated proteins and subsequent cell death [217].

It has been demonstrated that a series of plant-derived polyphenols, involving epigallocatechin gallate (EGCG), genistein, luteolin, apigenin, chrysin, quercetin, curcumin, and tannins, target UPS in cancer and improve chemotherapeutic responses [218,219,220,221,222,223]. EGCG inhibits the chymotrypsin-like activity of proteasome b5 subunit [170,224,225].

Combination of proteasome inhibitors with targeted therapies has been demonstrated to be promising for cancer treatment. Hydroxychloroquine, an autophagy inhibitor in combination with bortezomib, has been suggested as a promising strategy for the treatment of refractory and relapsed multiple myeloma [226,227]. Bortezomib in combination with monoclonal antibodies, daratumumab and elotuzumab, BCL-2 inhibitor venetoclax, and histone deacetylase (HDAC) inhibitor panobinostat shows synergistic effects in refractory and relapsed multiple myeloma [228,229,230,231,232]. Preclinical studies have shown that ACY-1215, an HDAC inhibitor, potentiates the activity of bortezomib against multiple myeloma cells [233,234]. Interestingly, TNBC cells have been shown to be sensitive to proteasome inhibitors, suggesting that proteasome inhibition may be an effective strategy for the treatment of TNBC patients [235]. The combination of lapatinib, a dual tyrosine kinase inhibitor with proteasome inhibitors, has been suggested to be promising for the treatment of TNBC patients [236].

In spite of the success for cancer treatment, bortezomib has been demonstrated to be associated with certain toxicities, involving peripheral neuropathy, hematologic toxicity, gastrointestinal toxicity, cardiovascular toxicity, and herpes zoster reactivation, indicating the adverse impacts of bortezomib on patients [237,238,239,240]. Additionally, cafilzomib has been shown to be associated with adverse cardiovascular toxicity [241,242,243].

Inhibitors for valosin-containing protein (VCP, also known as p97) ATPase that is responsible for the retrotranslocation of ERAD substrates include eeyarestatin, DbeQ, ML240, ML241, NMS-873, and CB-5083 [244,245,246,247,248,249,250]. Eeyarestatin has been demonstrated to activate ATF3 and ATF4 and to induce the expression of the pro-apoptotic NOXA in malignant melanoma cells, indicating its anti-cancer activity [251,252]. Additionally, CB-5083 has been shown to activate the UPR and to induce apoptosis in various hematological and solid tumors in vitro as well as in vivo [253,254,255].

### 6.5. Modulation of Chaperones

Modulation of chaperone has been suggested as a promising approach for cancer treatment. Given that BiP is closely associated with tumor stages as well as the therapeutic responses of cancers, BiP inhibitors have been identified and developed for the treatment of cancers [5]. Honokiol inhibits BiP and induces apoptosis in brain tumors [256]. Additionally, AB5 subtilase (SubAB) cytotoxin inhibits BiP by specific cleavage [257]. Interestingly, the recombinant form of the subtilase catalytic subunit (SubA) with human epidermal growth factor (EGF) for more enhanced action promotes a non-typical apoptosis when combined with photodynamic therapy [258,259]. Further, HA15 belonging to thiazole benzensulfonamides inhibits BiP and induces apoptosis in a variety of chemoresistant cancer cell lines in vitro as well as in vivo [260]. Interaction of HA15 with BiP leads to the dissociation of BiP from three arms of the UPR, thereby leading to the activation of the UPR signaling. Additionally, epigallocatechin-3-gallate (EGCG) has been shown to bind and inhibit the ATP-binding domain of BiP, thereby leading to the sensitization of glioma cells to chemotherapy [261]. Overexpressed BiP forms an inhibitory complex with caspase-7 and causes the inactivation of caspase-7, resulting in cancer progression and drug resistance. Interestingly, EGCG attenuates the complex formation of BiP and caspase-7, thereby preventing the anti-apoptotic effects of BiP. Versipelostatin downregulates the expression of BiP at the transcriptional levels and inhibits the expression of ATF4 and XBP1 [262]. In combination with cisplatin, versipelostatin inhibits BiP in stomach cancer xenograft [263].

ORP150 is an ER-resident HSP70 chaperone that is induced by ER stress as well as hypoxia [264]. Berberine, a natural alkaloid, has been shown to decrease the expression of ORP150 in liver cancer cell lines [265].

Geldanamycin targets GRP94, the ER resident homologue of HSP90, and induces apoptosis in B chronic lymphocytic leukemia cells [266,267]. OSU-03012, an inhibitor of GRP94 and GRP78, has been demonstrated to exhibit anti-cancer effects in combination with sildenafil, a well-known selective phosphodiesterase type 5 (PDE5) inhibitor [268,269]. 17-allylamino-17-demethoxygeldanamycin (17-AAG), a derivative of geldanamycin, binds to the amino-terminal ATP-binding domain of HSP90 and inhibits HSP90, resulting in cell death [270,271]. 17-AAG induces *XBP1* mRNA splicing and upregulates CHOP, thereby leading to cell death. Further, other HSP90 inhibitors, involving radicicol, SNX-2112, and retaspimycin, have been demonstrated to induce cell death via the activation of the UPR in cancer cells [272]. Interestingly, it has been shown that a combination of rapamycin with retaspimycin induces massive ER stress and regression of aggressive RAS-driven tumors [273]. Radamide, a chimeric compound containing quinone moiety from geldanamycin and resorcinol from radicicol, possesses high affinity for GRP94 and antiproliferative activities on a variety of cancer cell lines [274,275].

It has been demonstrated that PDIA1 inhibitors attenuate the pro-survival effects of the UPR in cancer and possess potent anti-cancer activity in melanoma and malignant glioma [276,277].

### 6.6. ER Stress and Immunogenic Cell Death

It has been demonstrated that anti-cancer agents, involving anthracyclines, bortezomib, and HDAC inhibitors and radiotherapy not only induce death of cancer cells, but also increase immunogenicity of cell death, thereby leading to the modulation of anti-tumor immunity in and around the tumor microenvironment [278,279,280,281]. This kind of cell death is referred to as immunogenic cell death (ICD). The immunogenicity of dying cells is delineated by the secretion or exposure of a variety of molecules, which is termed damage-associated molecular patterns (DAMPs). DAMPs include ATP secretion, passive release of high-mobility group box 1 (HMGB1), and surface exposure of calreticulin [110,281,282]. Once released from dying cells, DAMPs acquire pro-inflammatory and immunostimulatory activities, suggesting that DAMPs may transduce danger signals and activate immune systems to evoke anti-tumor immunity. In fact, a complex interconnection between autophagy, ER stress, and oxidative stress has been shown to regulate DAMPs [283,284,285,286,287]. DAMPs not only prime cancer-killing CD8+ T cells for the secretion of interferon γ (IFNγ), but also anti-cancer CD4+ T cells for the secretion of IFNγ and IL-17A [288].

PERK has been shown to be involved in the exposure of calreticulin in non-small-cell lung carcinomas (NSCLCs), thereby leading to ICD and anti-tumor immunity [289]. Furthermore, PERK activation by photodynamic therapy induces ATP secretion and the surface exposure of calreticulin, resulting in the clearance of human bladder carcinoma cells by DCs [283]. Interestingly, anthracyclines-induced ER stress promotes the partially active caspase-8-mediated cleavage of BAP31, which triggers the surface exposure of calreticulin at the plasma membrane and subsequent ICD [284]. Moreover, radiation and anthracycline treatment induce lethal ER stress, the excessive activation of the UPR, and an increase in the level of cytosolic Ca^2+^, thereby leading to the activation of inflammasome and ICD [290,291]. Therefore, ER stress-associated ICD might have pro-inflammatory and pro-immunological properties and combine physiological cell death with anti-tumor immunity, resulting in the induction of anti-cancer vaccine effect.

## 7. Conclusions and Future Perspectives

The UPR was classically demonstrated to be restricted to the maintenance of proteostasis in specialized secretory cells such as pancreatic β cells, plasma B cells, and salivary glands with the characteristics of accelerated protein synthesis and secretion and continuous generation of ER stress. However, it has been recently demonstrated that the UPR is also involved in a variety of physiological processes that are not restricted to protein synthesis and secretion, involving cell differentiation, inflammation, energy production, and lipid metabolism [39,292]. In the course of tumor development, tumor cells are continuously exposed to a variety of intrinsic as well as extrinsic perturbations, which result in ER stress and the subsequent activation of the UPR. The ability of tumor cells to restore homeostasis by resolving ER stress and to survive dominantly depends on the appropriate activation of the UPR, suggesting that the UPR is a central player in tumor development [293]. Furthermore, a variety of studies have revealed that tumors are “addicted” to the UPR. Intriguingly, not only the interaction of the UPR with other cellular processes, but also the crosstalk between ER stress and cell fate decisions via the communications of ER with mitochondria are pivotal for tumor development and therapeutic responses. Therefore, developing therapeutic strategies not only to modulate the UPR, but also to potentiate the crosstalk between ER stress and mitochondrial cell death have become desirable approaches of late.

Given that ER stress and the UPR is implicated in the etiology of cancer, the UPR could be potential therapeutic targets for cancer treatment. Inhibitors targeting the UPR of the ER have been successfully developed and shown to attenuate the growth of tumors alone or in combination with other pharmaceutical drugs and to reduce therapeutic resistance in combination with chemotherapeutic drugs in vivo. However, in spite of the success of proteasome inhibition in multiple myeloma, many patients have shown to develop resistance to the proteasome inhibitors. Furthermore, the disadvantage of bortezomib is its high toxicity [294,295]. Additionally, targeting the UPR has been demonstrated to have unpredictable side effects, mainly due to the opposing pro- and anti-survival roles of the UPR. Further, inhibition of one arm of the UPR may result in the alteration of the other arms of the UPR as well as other pathways in tumors, conferring adverse effects on cancer treatment. In conclusion, identification of novel molecules and mechanisms that are involved in the activation and persistence of the UPR in cancer can help us understanding how organisms cope with ER stress and developing new therapeutic strategies. Furthermore, it could be promising to develop novel strategies targeting cancer-intrinsic defects in combination with the UPR-targeting therapy for cancer treatment.

## References

[1] Alberts B., Johnson A., Lewis J., Raff M., Roberts K., Walter P. (2002). The endoplasmic reticulum. Molecular Biology of the Cell.

[2] Görlach A., Klappa P., Kietzmann D.T. (2006). The endoplasmic reticulum: Folding, calcium homeostasis, signaling, and redox control. Antioxid. Redox Signal..

[3] Berridge M.J., Lipp P., Bootman M.D. (2000). The versatility and universality of calcium signalling. Nat. Rev. Mol. Cell Biol..

[4] Oakes S.A., Papa F.R. (2015). The role of endoplasmic reticulum stress in human pathology. Annu. Rev. Pathol. Mech. Dis..

[5] Wang M., Kaufman R.J. (2014). The impact of the endoplasmic reticulum protein-folding environment on cancer development. Nat. Rev. Cancer.

[6] Schröder M., Kaufman R.J. (2005). The mammalian unfolded protein response. Annu. Rev. Biochem..

[7] López-Otín C., Blasco M.A., Partridge L., Serrano M., Kroemer G. (2013). The hallmarks of aging. Cell.

[8] Chiti F., Dobson C.M. (2006). Protein misfolding, functional amyloid, and human disease. Annu. Rev. Biochem..

[9] Wang M., Kaufman R.J. (2016). Protein misfolding in the endoplasmic reticulum as a conduit to human disease. Nature.

[10] Schröder M. (2008). Endoplasmic reticulum stress responses. Cell. Mol. Life Sci..

[11] Walter P., Ron D. (2011). The unfolded protein response: From stress pathway to homeostatic regulation. Science.

[12] Moon H.W., Han H.G., Jeon Y.J. (2018). Protein quality control in the endoplasmic reticulum and cancer. Int. J. Mol. Sci..

[13] Yoo Y.S., Han H.G., Jeon Y.J. (2017). Unfolded protein response of the endoplasmic reticulum in tumor progression and immunogenicity. Oxidative Med. Cell. Longev..

[14] Bertolotti A., Wang X., Novoa I., Jungreis R., Schlessinger K., Cho J.H., West A.B., Ron D. (2001). Increased sensitivity to dextran sodium sulfate colitis in ire1β-deficient mice. J. Clin. Investig..

[15] Otero J.H., Lizák B., Hendershot L.M. (2010). Life and death of a bip substrate. Semin. Cell Dev. Biol..

[16] Bertolotti A., Zhang Y., Hendershot L.M., Harding H.P., Ron D. (2000). Dynamic interaction of Bip and ER stress transducers in the unfolded-protein response. Nat. Cell Biol..

[17] Pincus D., Chevalier M.W., Aragón T., Van Anken E., Vidal S.E., El-Samad H., Walter P. (2010). Bip binding to the ER-stress sensor ire1 tunes the homeostatic behavior of the unfolded protein response. PLoS Biol..

[18] Groenendyk J., Peng Z., Dudek E., Fan X., Mizianty M.J., Dufey E., Urra H., Sepulveda D., Rojas-Rivera D., Lim Y. (2014). Interplay between the oxidoreductase pdia6 and microrna-322 controls the response to disrupted endoplasmic reticulum calcium homeostasis. Sci. Signal..

[19] Eletto D., Eletto D., Dersh D., Gidalevitz T., Argon Y. (2014). Protein disulfide isomerase a6 controls the decay of ire1α signaling via disulfide-dependent association. Mol. Cell.

[20] Higa A., Taouji S., Lhomond S., Jensen D., Fernandez-Zapico M.E., Simpson J.C., Pasquet J.M., Schekman R., Chevet E. (2014). Endoplasmic reticulum stress-activated transcription factor atf6α requires the disulfide isomerase pdia5 to modulate chemoresistance. Mol. Cell. Biol..

[21] Gardner B.M., Walter P. (2011). Unfolded proteins are ire1-activating ligands that directly induce the unfolded protein response. Science.

[22] Korennykh A.V., Egea P.F., Korostelev A.A., Finer-Moore J., Zhang C., Shokat K.M., Stroud R.M., Walter P. (2009). The unfolded protein response signals through high-order assembly of ire1. Nature.

[23] Li H., Korennykh A.V., Behrman S.L., Walter P. (2010). Mammalian endoplasmic reticulum stress sensor ire1 signals by dynamic clustering. Proc. Natl. Acad. Sci. USA.

[24] Harding H.P., Zhang Y., Ron D. (1999). Protein translation and folding are coupled by an endoplasmic-reticulum-resident kinase. Nature.

[25] Harding H.P., Zhang Y., Zeng H., Novoa I., Lu P.D., Calfon M., Sadri N., Yun C., Popko B., Paules R. (2003). An integrated stress response regulates amino acid metabolism and resistance to oxidative stress. Mol. Cell.

[26] Vattem K.M., Wek R.C. (2004). Reinitiation involving upstream ORFs regulates ATF4 mRNA translation in mammalian cells. Proc. Natl. Acad. Sci. USA.

[27] Marciniak S.J., Yun C.Y., Oyadomari S., Novoa I., Zhang Y., Jungreis R., Nagata K., Harding H.P., Ron D. (2004). Chop induces death by promoting protein synthesis and oxidation in the stressed endoplasmic reticulum. Genes Dev..

[28] Cullinan S.B., Diehl J.A. (2006). Coordination of ER and oxidative stress signaling: The PERK/Nrf2 signaling pathway. Int. J. Biochem. Cell Biol..

[29] Tirasophon W., Welihinda A.A., Kaufman R.J. (1998). A stress response pathway from the endoplasmic reticulum to the nucleus requires a novel bifunctional protein kinase/endoribonuclease (ire1p) in mammalian cells. Genes Dev..

[30] Sepulveda D., Rojas-Rivera D., Rodriguez D.A., Groenendyk J., Köhler A., Lebeaupin C., Ito S., Urra H., Carreras-Sureda A., Hazari Y. (2018). Interactome screening identifies the ER luminal chaperone hsp47 as a regulator of the unfolded protein response transducer IRE1α. Mol. Cell.

[31] Lamriben L., Hebert D.N. (2018). Activating and repressing IRE1α: The Hsp47 and Bip Tug of War. Mol. Cell.

[32] Yoshida H., Matsui T., Yamamoto A., Okada T., Mori K. (2001). Xbp1 mrna is induced by atf6 and spliced by ire1 in response to ER stress to produce a highly active transcription factor. Cell.

[33] Liu Y., Adachi M., Zhao S., Hareyama M., Koong A., Luo D., Rando T., Imai K., Shinomura Y. (2009). Preventing oxidative stress: A new role for XBP1. Cell Death Differ..

[34] Lee A.H., Iwakoshi N.N., Glimcher L.H. (2003). Xbp-1 regulates a subset of endoplasmic reticulum resident chaperone genes in the unfolded protein response. Mol. Cell. Biol..

[35] Haze K., Yoshida H., Yanagi H., Yura T., Mori K. (1999). Mammalian transcription factor atf6 is synthesized as a transmembrane protein and activated by proteolysis in response to endoplasmic reticulum stress. Mol. Biol. Cell.

[36] Lee K., Tirasophon W., Shen X., Michalak M., Prywes R., Okada T., Yoshida H., Mori K., Kaufman R.J. (2002). IRE1-mediated unconventional mRNA splicing and S2P-mediated ATF6 cleavage merge to regulate XBP1 in signaling the unfolded protein response. Genes Dev..

[37] Diedrich K., Fauser B.C.J.M., Devroey P., Griesinger G. (2007). The role of the endometrium and embryo in human implantation. Hum. Reprod. Update.

[38] Bommiasamy H., Back S.H., Fagone P., Lee K., Meshinchi S., Vink E., Sriburi R., Frank M., Jackowski S., Kaufman R.J. (2009). Atf6α induces xbp1-independent expansion of the endoplasmic reticulum. J. Cell Sci..

[39] Rutkowski D.T., Hegde R.S. (2010). Regulation of basal cellular physiology by the homeostatic unfolded protein response. J. Cell Biol..

[40] Bernhard W., Rouiller C. (1956). Close topographical relationship between mitochondria and ergastoplasm of liver cells in a definite phase of cellular activity. J. Cell Biol..

[41] Sassano M.L., van Vliet A.R., Agostinis P. (2017). Mitochondria-associated membranes as networking platforms and regulators of cancer cell fate. Front. Oncol..

[42] Doghman-Bouguerra M., Lalli E. (2019). Er-mitochondria interactions: Both strength and weakness within cancer cells. Biochim. Biophys. Acta BBA Mol. Cell Res..

[43] Cárdenas C., Miller R.A., Smith I., Bui T., Molgó J., Müller M., Vais H., Cheung K.H., Yang J., Parker I. (2010). Essential regulation of cell bioenergetics by constitutive InsP3 receptor Ca^2+^ transfer to mitochondria. Cell.

[44] van Vliet A.R., Verfaillie T., Agostinis P. (2014). New functions of mitochondria associated membranes in cellular signaling. Biochim. Biophys. Acta BBA Mol. Cell Res..

[45] van Vliet A.R., Agostinis P. (2016). When under pressure, get closer: Perking up membrane contact sites during ER stress. Biochem. Soc. Trans..

[46] Carreras-Sureda A., Pihán P., Hetz C. (2017). The unfolded protein response: At the intersection between endoplasmic reticulum function and mitochondrial bioenergetics. Front. Oncol..

[47] Gutiérrez T., Simmen T. (2018). Endoplasmic reticulum chaperones tweak the mitochondrial calcium rheostat to control metabolism and cell death. Cell Calcium.

[48] Hayashi T., Su T.P. (2007). Sigma-1 receptor chaperones at the ER-mitochondrion interface regulate Ca^2+^ signaling and cell survival. Cell.

[49] Verfaillie T., Rubio N., Garg A., Bultynck G., Rizzuto R., Decuypere J., Piette J., Linehan C., Gupta S., Samali A. (2012). Perk is required at the ER-mitochondrial contact sites to convey apoptosis after ROS-based ER stress. Cell Death Differ..

[50] Chami M., Oulès B., Szabadkai G., Tacine R., Rizzuto R., Paterlini-Bréchot P. (2008). Role of serca1 truncated isoform in the proapoptotic calcium transfer from ER to mitochondria during ER stress. Mol. Cell.

[51] Calì T., Ottolini D., Negro A., Brini M. (2013). Enhanced parkin levels favor ER-mitochondria crosstalk and guarantee Ca^2+^ transfer to sustain cell bioenergetics. Biochim. Biophys. Acta BBA Mol. Basis Dis..

[52] Urra H., Dufey E., Lisbona F., Rojas-Rivera D., Hetz C. (2013). When ER stress reaches a dead end. Biochim. Biophys. Acta BBA Mol. Cell Res..

[53] Misra U.K., Deedwania R., Pizzo S.V. (2006). Activation and cross-talk between Akt, Nf-κB, and unfolded protein response signaling in 1-LN prostate cancer cells consequent to ligation of cell surface-associated GRP78. J. Biol. Chem..

[54] Burikhanov R., Zhao Y., Goswami A., Qiu S., Schwarze S.R., Rangnekar V.M. (2009). The tumor suppressor Par-4 activates an extrinsic pathway for apoptosis. Cell.

[55] Davidson D.J., Haskell C., Majest S., Kherzai A., Egan D.A., Walter K.A., Schneider A., Gubbins E.F., Solomon L., Chen Z. (2005). Kringle 5 of human plasminogen induces apoptosis of endothelial and tumor cells through surface-expressed glucose-regulated protein 78. Cancer Res..

[56] Itoh K., Chiba T., Takahashi S., Ishii T., Igarashi K., Katoh Y., Oyake T., Hayashi N., Satoh K., Hatayama I. (1997). An Nrf2/small Maf heterodimer mediates the induction of phase ii detoxifying enzyme genes through antioxidant response elements. Biochem. Biophys. Res. Commun..

[57] Kensler T.W., Wakabayashi N., Biswal S. (2007). Cell survival responses to environmental stresses via the keap1-Nrf2-are pathway. Annu. Rev. Pharmacol. Toxicol..

[58] Niture S.K., Jaiswal A.K. (2012). Nrf2 protein up-regulates antiapoptotic protein Bcl-2 and prevents cellular apoptosis. J. Biol. Chem..

[59] Niture S.K., Jaiswal A.K. (2013). Nrf2-induced antiapoptotic Bcl-xL protein enhances cell survival and drug resistance. Free Radic. Biol. Med..

[60] Chitnis N.S., Pytel D., Bobrovnikova-Marjon E., Pant D., Zheng H., Maas N.L., Frederick B., Kushner J.A., Chodosh L.A., Koumenis C. (2012). Mir-211 is a prosurvival microrna that regulates chop expression in a perk-dependent manner. Mol. Cell.

[61] Lin J.H., Li H., Zhang Y., Ron D., Walter P. (2009). Divergent effects of perk and ire1 signaling on cell viability. PLoS ONE.

[62] Song B., Scheuner D., Ron D., Pennathur S., Kaufman R.J. (2008). Chop deletion reduces oxidative stress, improves β cell function, and promotes cell survival in multiple mouse models of diabetes. J. Clin. Investig..

[63] Woo C.W., Kutzler L., Kimball S.R., Tabas I. (2012). Toll-like receptor activation suppresses ER stress factor CHOP and translation inhibition through activation of eiF2B. Nat. Cell Biol..

[64] Han J., Back S.H., Hur J., Lin Y.H., Gildersleeve R., Shan J., Yuan C.L., Krokowski D., Wang S., Hatzoglou M. (2013). Er-stress-induced transcriptional regulation increases protein synthesis leading to cell death. Nat. Cell Biol..

[65] Brush M.H., Weiser D.C., Shenolikar S. (2003). Growth arrest and DNA damage-inducible protein GADD34 targets protein phosphatase 1α to the endoplasmic reticulum and promotes dephosphorylation of the α subunit of eukaryotic translation initiation factor 2. Mol. Cell. Biol..

[66] Kaufman R.J., Malhotra J.D. (2014). Calcium trafficking integrates endoplasmic reticulum function with mitochondrial bioenergetics. Biochim. Biophys. Acta BBA Mol. Cell Res..

[67] McCullough K.D., Martindale J.L., Klotz L.O., Aw T.Y., Holbrook N.J. (2001). GADD153 sensitizes cells to endoplasmic reticulum stress by down-regulating bcl2 and perturbing the cellular redox state. Mol. Cell. Biol..

[68] Emily H.Y.C., Wei M.C., Weiler S., Flavell R.A., Mak T.W., Lindsten T., Korsmeyer S.J. (2001). BCL-2, BCL-XL sequester BH3 domain-only molecules preventing BAX-and BAK-mediated mitochondrial apoptosis. Mol. Cell.

[69] Rodriguez D., Rojas-Rivera D., Hetz C. (2011). Integrating stress signals at the endoplasmic reticulum: The BCL-2 protein family rheostat. Biochim. Biophys. Acta BBA Mol. Cell Res..

[70] Galehdar Z., Swan P., Fuerth B., Callaghan S.M., Park D.S., Cregan S.P. (2010). Neuronal apoptosis induced by endoplasmic reticulum stress is regulated by ATF4–CHOP-mediated induction of the Bcl-2 homology 3-only member puma. J. Neurosci..

[71] Puthalakath H., O’Reilly L.A., Gunn P., Lee L., Kelly P.N., Huntington N.D., Hughes P.D., Michalak E.M., McKimm-Breschkin J., Motoyama N. (2007). ER stress triggers apoptosis by activating BH3-only protein Bim. Cell.

[72] Yamaguchi H., Wang H.G. (2004). CHOP is involved in endoplasmic reticulum stress-induced apoptosis by enhancing DR5 expression in human carcinoma cells. J. Biol. Chem..

[73] Du K., Herzig S., Kulkarni R.N., Montminy M. (2003). Trb3: A tribbles homolog that inhibits AKT/PKB activation by insulin in liver. Science.

[74] Grivennikov S.I., Karin M. (2010). Dangerous liaisons: Stat3 and NF-κB collaboration and crosstalk in cancer. Cytokine Growth Factor Rev..

[75] Woehlbier U., Hetz C. (2011). Modulating stress responses by the uprosome: A matter of life and death. Trends Biochem. Sci..

[76] Hetz C., Glimcher L.H. (2009). Fine-tuning of the unfolded protein response: Assembling the ire1α interactome. Mol. Cell.

[77] Lisbona F., Hetz C. (2009). Turning off the Unfolded Protein Response: An Interplay between the Apoptosis Machinery and ER Stress Signaling.

[78] Hetz C., Bernasconi P., Fisher J., Lee A.H., Bassik M.C., Antonsson B., Brandt G.S., Iwakoshi N.N., Schinzel A., Glimcher L.H. (2006). Proapoptotic BAX and BAK modulate the unfolded protein response by a direct interaction with IRE1α. Science.

[79] Luo D., He Y., Zhang H., Yu L., Chen H., Xu Z., Tang S., Urano F., Min W. (2008). AIP1 is critical in transducing IRE1-mediated endoplasmic reticulum stress response. J. Biol. Chem..

[80] Gu F., Nguyên D.T., Stuible M., Dubé N., Tremblay M.L., Chevet E. (2004). Protein-tyrosine phosphatase 1B potentiates IRE1 signaling during endoplasmic reticulum stress. J. Biol. Chem..

[81] Gupta S., Deepti A., Deegan S., Lisbona F., Hetz C., Samali A. (2010). HSP72 protects cells from ER stress-induced apoptosis via enhancement of IRE1Α-XBP1 signaling through a physical interaction. PLoS Biol..

[82] Urano F., Wang X., Bertolotti A., Zhang Y., Chung P., Harding H.P., Ron D. (2000). Coupling of stress in the ER to activation of JNK protein kinases by transmembrane protein kinase IRE1. Science.

[83] Nishitoh H., Matsuzawa A., Tobiume K., Saegusa K., Takeda K., Inoue K., Hori S., Kakizuka A., Ichijo H. (2002). ASK1 is essential for endoplasmic reticulum stress-induced neuronal cell death triggered by expanded polyglutamine repeats. Genes Dev..

[84] Yoneda T., Imaizumi K., Oono K., Yui D., Gomi F., Katayama T., Tohyama M. (2001). Activation of caspase-12, an endoplastic reticulum (ER) resident caspase, through tumor necrosis factor receptor-associated factor 2-dependent mechanism in response to the ER stress. J. Biol. Chem..

[85] Szegezdi E., Logue S.E., Gorman A.M., Samali A. (2006). Mediators of endoplasmic reticulum stress-induced apoptosis. EMBO Rep..

[86] Szegezdi E., MacDonald D.C., Ní Chonghaile T.O., Gupta S., Samali A. (2009). BCL-2 family on guard at the ER. Am. J. Physiol. Cell Physiol..

[87] Klee M., Pallauf K., Alcalá S., Fleischer A., Pimentel-Muiños F.X. (2009). Mitochondrial apoptosis induced by BH3-only molecules in the exclusive presence of endoplasmic reticular Bak. EMBO J..

[88] Lisbona F., Rojas-Rivera D., Thielen P., Zamorano S., Todd D., Martinon F., Glavic A., Kress C., Lin J.H., Walter P. (2009). Bax inhibitor-1 is a negative regulator of the ER stress sensor ire1α. Mol. Cell.

[89] Lee G.H., Kim H.K., Chae S.W., Kim D.S., Ha K.C., Cuddy M., Kress C., Reed J.C., Kim H.R., Chae H.J. (2007). Bax inhibitor-1 regulates endoplasmic reticulum stress-associated reactive oxygen species and heme oxygenase-1 expression. J. Biol. Chem..

[90] Bailly-Maitre B., Belgardt B.F., Jordan S.D., Coornaert B., von Freyend M.J., Kleinridders A., Mauer J., Cuddy M., Kress C.L., Willmes D. (2010). Hepatic bax inhibitor-1 inhibits IRE1α and protects from obesity-associated insulin resistance and glucose intolerance. J. Biol. Chem..

[91] Bailly-Maitre B., Fondevila C., Kaldas F., Droin N., Luciano F., Ricci J.E., Croxton R., Krajewska M., Zapata J.M., Kupiec-Weglinski J.W. (2006). Cytoprotective gene bi-1 is required for intrinsic protection from endoplasmic reticulum stress and ischemia-reperfusion injury. Proc. Natl. Acad. Sci. USA.

[92] Rong J., Chen L., Toth J.I., Tcherpakov M., Petroski M.D., Reed J.C. (2011). Bifunctional apoptosis regulator (bar), an endoplasmic reticulum (ER)-associated E3 ubiquitin ligase, modulates BI-1 protein stability and function in ER stress. J. Biol. Chem..

[93] Hollien J., Weissman J.S. (2006). Decay of endoplasmic reticulum-localized mRNAS during the unfolded protein response. Science.

[94] Han D., Lerner A.G., Walle L.V., Upton J.P., Xu W., Hagen A., Backes B.J., Oakes S.A., Papa F.R. (2009). Ire1α kinase activation modes control alternate endoribonuclease outputs to determine divergent cell fates. Cell.

[95] Hayashi S., Wakasa Y., Ozawa K., Takaiwa F. (2016). Characterization of IRE 1 ribonuclease-mediated mRNA decay in plants using transient expression analyses in rice protoplasts. New Phytol..

[96] Oikawa D., Tokuda M., Hosoda A., Iwawaki T. (2010). Identification of a consensus element recognized and cleaved by IRE1α. Nucleic Acids Res..

[97] Ghosh R., Wang L., Wang E.S., Perera B.G.K., Igbaria A., Morita S., Prado K., Thamsen M., Caswell D., Macias H. (2014). Allosteric inhibition of the IRE1α RNase preserves cell viability and function during endoplasmic reticulum stress. Cell.

[98] Lerner A.G., Upton J.P., Praveen P., Ghosh R., Nakagawa Y., Igbaria A., Shen S., Nguyen V., Backes B.J., Heiman M. (2012). IRE1α induces thioredoxin-interacting protein to activate the NLRP3 inflammasome and promote programmed cell death under irremediable ER stress. Cell Metab..

[99] Upton J.P., Wang L., Han D., Wang E.S., Huskey N.E., Lim L., Truitt M., McManus M.T., Ruggero D., Goga A. (2012). IRE1α cleaves select micrornas during ER stress to derepress translation of proapoptotic caspase-2. Science.

[100] Shiraishi H., Okamoto H., Yoshimura A., Yoshida H. (2006). ER stress-induced apoptosis and caspase-12 activation occurs downstream of mitochondrial apoptosis involving apaf-1. J. Cell Sci..

[101] Saleh M., Mathison J.C., Wolinski M.K., Bensinger S.J., Fitzgerald P., Droin N., Ulevitch R.J., Green D.R., Nicholson D.W. (2006). Enhanced bacterial clearance and sepsis resistance in caspase-12-deficient mice. Nature.

[102] Nakagawa T., Zhu H., Morishima N., Li E., Xu J., Yankner B.A., Yuan J. (2000). Caspase-12 mediates endoplasmic-reticulum-specific apoptosis and cytotoxicity by amyloid-β. Nature.

[103] Lamkanfi M., Kalai M., Vandenabeele P. (2004). Caspase-12: An overview. Cell Death Differ..

[104] Kim S.J., Zhang Z., Hitomi E., Lee Y.C., Mukherjee A.B. (2006). Endoplasmic reticulum stress-induced caspase-4 activation mediates apoptosis and neurodegeneration in incl. Hum. Mol. Genet..

[105] Hitomi J., Katayama T., Eguchi Y., Kudo T., Taniguchi M., Koyama Y., Manabe T., Yamagishi S., Bando Y., Imaizumi K. (2004). Involvement of caspase-4 in endoplasmic reticulum stress-induced apoptosis and aβ-induced cell death. J. Cell Biol..

[106] Breckenridge D.G., Stojanovic M., Marcellus R.C., Shore G.C. (2003). Caspase cleavage product of BAP31 induces mitochondrial fission through endoplasmic reticulum calcium signals, enhancing cytochrome c release to the cytosol. J. Cell Biol..

[107] Hanahan D., Weinberg R.A. (2011). Hallmarks of cancer: The next generation. Cell.

[108] Ma Y., Hendershot L.M. (2004). The role of the unfolded protein response in tumour development: Friend or foe?. Nat. Rev. Cancer.

[109] Urra H., Dufey E., Avril T., Chevet E., Hetz C. (2016). Endoplasmic reticulum stress and the hallmarks of cancer. Trends Cancer.

[110] Vanacker H., Vetters J., Moudombi L., Caux C., Janssens S., Michallet M.C. (2017). Emerging role of the unfolded protein response in tumor immunosurveillance. Trends Cancer.

[111] Volmer R., Ron D. (2015). Lipid-dependent regulation of the unfolded protein response. Curr. Opin. Cell Biol..

[112] Clarke H.J., Chambers J.E., Liniker E., Marciniak S.J. (2014). Endoplasmic reticulum stress in malignancy. Cancer Cell.

[113] Andruska N., Zheng X., Yang X., Helferich W.G., Shapiro D.J. (2015). Anticipatory estrogen activation of the unfolded protein response is linked to cell proliferation and poor survival in estrogen receptor α-positive breast cancer. Oncogene.

[114] Dong D., Ni M., Li J., Xiong S., Ye W., Virrey J.J., Mao C., Ye R., Wang M., Pen L. (2008). Critical role of the stress chaperone GRP78/BiP in tumor proliferation, survival, and tumor angiogenesis in transgene-induced mammary tumor development. Cancer Res..

[115] Fu Y., Wey S., Wang M., Ye R., Liao C.P., Roy-Burman P., Lee A.S. (2008). Pten null prostate tumorigenesis and AKT activation are blocked by targeted knockout of ER chaperone GRP78/BiP in prostate epithelium. Proc. Natl. Acad. Sci. USA.

[116] Luo B., Lee A.S. (2013). The critical roles of endoplasmic reticulum chaperones and unfolded protein response in tumorigenesis and anticancer therapies. Oncogene.

[117] Lee A.S. (2007). GRP78 induction in cancer: Therapeutic and prognostic implications. Cancer Res..

[118] Verfaillie T., Garg A.D., Agostinis P. (2013). Targeting ER stress induced apoptosis and inflammation in cancer. Cancer Lett..

[119] Lee A.S. (2014). Glucose-regulated proteins in cancer: Molecular mechanisms and therapeutic potential. Nat. Rev. Cancer.

[120] Jamora C., Dennert G., Lee A.S. (1996). Inhibition of tumor progression by suppression of stress protein GRP78/BiP induction in fibrosarcoma B/C10ME. Proc. Natl. Acad. Sci. USA.

[121] Wang G., Yang Z.Q., Zhang K. (2010). Endoplasmic reticulum stress response in cancer: Molecular mechanism and therapeutic potential. Am. J. Transl. Res..

[122] Mintz P.J., Kim J., Do K.A., Wang X., Zinner R.G., Cristofanilli M., Arap M.A., Hong W.K., Troncoso P., Logothetis C.J. (2003). Fingerprinting the circulating repertoire of antibodies from cancer patients. Nat. Biotechnol..

[123] Denoyelle C., Abou-Rjaily G., Bezrookove V., Verhaegen M., Johnson T.M., Fullen D.R., Pointer J.N., Gruber S.B., Su L.D., Nikiforov M.A. (2006). Anti-oncogenic role of the endoplasmic reticulum differentially activated by mutations in the mapk pathway. Nat. Cell Biol..

[124] Uramoto H., Sugio K., Oyama T., Nakata S., Ono K., Yoshimastu T., Morita M., Yasumoto K. (2005). Expression of endoplasmic reticulum molecular chaperone grp78 in human lung cancer and its clinical significance. Lung Cancer.

[125] Hsu W.M., Hsieh F.J., Jeng Y.M., Kuo M.L., Tsao P.N., Lee H., Lin M.T., Lai H.S., Chen C.N., Lai D.M. (2005). GRP78 expression correlates with histologic differentiation and favorable prognosis in neuroblastic tumors. Int. J. Cancer.

[126] Xu G., Liu K., Anderson J., Patrene K., Lentzsch S., Roodman G.D., Ouyang H. (2012). Expression of XBP1s in bone marrow stromal cells is critical for myeloma cell growth and osteoclast formation. Blood.

[127] Fujimoto T., Onda M., Nagai H., Nagahata T., Ogawa K., Emi M. (2003). Upregulation and overexpression of human X-box binding protein 1 (hXBP-1) gene in primary breast cancers. Breast Cancer.

[128] Shuda M., Kondoh N., Imazeki N., Tanaka K., Okada T., Mori K., Hada A., Arai M., Wakatsuki T., Matsubara O. (2003). Activation of the atf6, XBP1 and GRP78 genes in human hepatocellular carcinoma: A possible involvement of the ER stress pathway in hepatocarcinogenesis. J. Hepatol..

[129] Sun H., Lin D.C., Guo X., Masouleh B.K., Gery S., Cao Q., Alkan S., Ikezoe T., Akiba C., Paquette R. (2016). Inhibition of IRE1α-driven pro-survival pathways is a promising therapeutic application in acute myeloid leukemia. Oncotarget.

[130] Chen X., Iliopoulos D., Zhang Q., Tang Q., Greenblatt M.B., Hatziapostolou M., Lim E., Tam W.L., Ni M., Chen Y. (2014). XBP1 promotes triple-negative breast cancer by controlling the HIF1α pathway. Nature.

[131] McGrath E.P., Logue S.E., Mnich K., Deegan S., Jäger R., Gorman A.M., Samali A. (2018). The unfolded protein response in breast cancer. Cancers.

[132] Zhao N., Cao J., Xu L., Tang Q., Dobrolecki L.E., Lv X., Talukdar M., Lu Y., Wang X., Hu D.Z. (2018). Pharmacological targeting of MYC-regulated IRE1/XBP1 pathway suppresses MYC-driven breast cancer. J. Clin. Investig..

[133] Logue S.E., McGrath E.P., Cleary P., Greene S., Mnich K., Almanza A., Chevet E., Dwyer R.M., Oommen A., Legembre P. (2018). Inhibition of ire1 RNase activity modulates the tumor cell secretome and enhances response to chemotherapy. Nat. Commun..

[134] Gupta A., Hossain M.M., Miller N., Kerin M., Callagy G., Gupta S. (2016). NCOA3 coactivator is a transcriptional target of XBP1 and regulates PERK–eIF2α–ATF4 signalling in breast cancer. Oncogene.

[135] Bagratuni T., Wu P., de Castro D.G., Davenport E.L., Dickens N.J., Walker B.A., Boyd K., Johnson D.C., Gregory W., Morgan G.J. (2010). XBP1s levels are implicated in the biology and outcome of myeloma mediating different clinical outcomes to thalidomide-based treatments. Blood.

[136] Greenman C., Stephens P., Smith R., Dalgliesh G.L., Hunter C., Bignell G., Davies H., Teague J., Butler A., Stevens C. (2007). Patterns of somatic mutation in human cancer genomes. Nature.

[137] Xue Z., He Y., Ye K., Gu Z., Mao Y., Qi L. (2011). A conserved structural determinant located at the interdomain region of mammalian inositol-requiring enzyme 1α. J. Biol. Chem..

[138] Pluquet O., Dejeans N., Bouchecareilh M., Lhomond S., Pineau R., Higa A., Delugin M., Combe C., Loriot S., Cubel G. (2013). Posttranscriptional regulation of per1 underlies the oncogenic function of ireα. Cancer Res..

[139] Masouleh B.K., Geng H., Hurtz C., Chan L.N., Logan A.C., Chang M.S., Huang C., Swaminathan S., Sun H., Paietta E. (2014). Mechanistic rationale for targeting the unfolded protein response in pre-b acute lymphoblastic leukemia. Proc. Natl. Acad. Sci. USA.

[140] Mimura N., Fulciniti M., Gorgun G., Tai Y.T., Cirstea D., Santo L., Hu Y., Fabre C., Minami J., Ohguchi H. (2012). Blockade of XBP1 splicing by inhibition of IRE1α is a promising therapeutic option in multiple myeloma. Blood.

[141] Papandreou I., Denko N.C., Olson M., Van Melckebeke H., Lust S., Tam A., Solow-Cordero D.E., Bouley D.M., Offner F., Niwa M. (2011). Identification of an ire1alpha endonuclease specific inhibitor with cytotoxic activity against human multiple myeloma. Blood.

[142] Bi M., Naczki C., Koritzinsky M., Fels D., Blais J., Hu N., Harding H., Novoa I., Varia M., Raleigh J. (2005). ER stress-regulated translation increases tolerance to extreme hypoxia and promotes tumor growth. EMBO J..

[143] Blais J.D., Addison C.L., Edge R., Falls T., Zhao H., Wary K., Koumenis C., Harding H.P., Ron D., Holcik M. (2006). Perk-dependent translational regulation promotes tumor cell adaptation and angiogenesis in response to hypoxic stress. Mol. Cell. Biol..

[144] Dey S., Sayers C.M., Verginadis I.I., Lehman S.L., Cheng Y., Cerniglia G.J., Tuttle S.W., Feldman M.D., Zhang P.J., Fuchs S.Y. (2015). Atf4-dependent induction of heme oxygenase 1 prevents anoikis and promotes metastasis. J. Clin. Investig..

[145] Cullinan S.B., Zhang D., Hannink M., Arvisais E., Kaufman R.J., Diehl J.A. (2003). Nrf2 is a direct perk substrate and effector of PERK-dependent cell survival. Mol. Cell. Biol..

[146] Del Vecchio C.A., Feng Y., Sokol E.S., Tillman E.J., Sanduja S., Reinhardt F., Gupta P.B. (2014). De-differentiation confers multidrug resistance via noncanonical PERK-Nrf2 signaling. PLoS Biol..

[147] Pytel D., Majsterek I., Diehl J.A. (2016). Tumor progression and the different faces of the PERK kinase. Oncogene.

[148] Wang Y., Alam G.N., Ning Y., Visioli F., Dong Z., Nör J.E., Polverini P.J. (2012). The unfolded protein response induces the angiogenic switch in human tumor cells through the PERK/ATF4 pathway. Cancer Res..

[149] Chang K.C., Chen P.C.H., Chen Y.P., Chang Y., Su I.J. (2011). Dominant expression of survival signals of endoplasmic reticulum stress response in hodgkin lymphoma. Cancer Sci..

[150] Karali E., Bellou S., Stellas D., Klinakis A., Murphy C., Fotsis T. (2014). VEGF signals through ATF6 and PERK to promote endothelial cell survival and angiogenesis in the absence of ER stress. Mol. Cell.

[151] Aguirre-Ghiso J.A. (2007). Models, mechanisms and clinical evidence for cancer dormancy. Nat. Rev. Cancer.

[152] Páez D., Labonte M.J., Bohanes P., Zhang W., Benhanim L., Ning Y., Wakatsuki T., Loupakis F., Lenz H.J. (2012). Cancer dormancy: A model of early dissemination and late cancer recurrence. Clin. Cancer Res..

[153] Schewe D.M., Aguirre-Ghiso J.A. (2008). ATF6α-Rheb-mTOR signaling promotes survival of dormant tumor cells in vivo. Proc. Natl. Acad. Sci. USA.

[154] Ginos M.A., Page G.P., Michalowicz B.S., Patel K.J., Volker S.E., Pambuccian S.E., Ondrey F.G., Adams G.L., Gaffney P.M. (2004). Identification of a gene expression signature associated with recurrent disease in squamous cell carcinoma of the head and neck. Cancer Res..

[155] Chang Y., Tseng C., Wang M., Chang W.C., Lee C., Chen L., Hung M.C., Su J.L. (2016). Deacetylation of HSPA5 by HDAC6 leads to GP78-mediated HSPA5 ubiquitination at K447 and suppresses metastasis of breast cancer. Oncogene.

[156] Chang Y.W., Chen H.A., Tseng C.F., Hong C.C., Ma J.T., Hung M.C., Wu C.H., Huang M.T., Su J.L. (2014). De-acetylation and degradation of HSPA5 is critical for E1A metastasis suppression in breast cancer cells. Oncotarget.

[157] Auf G., Jabouille A., Guérit S., Pineau R., Delugin M., Bouchecareilh M., Magnin N., Favereaux A., Maitre M., Gaiser T. (2010). Inositol-requiring enzyme 1α is a key regulator of angiogenesis and invasion in malignant glioma. Proc. Natl. Acad. Sci. USA.

[158] Dejeans N., Pluquet O., Lhomond S., Grise F., Bouchecareilh M., Juin A., Meynard-Cadars M., Bidaud-Meynard A., Gentil C., Moreau V. (2012). Autocrine control of glioma cells adhesion and migration through IRE1α-mediated cleavage of sparc mrna. J. Cell Sci..

[159] Cubillos-Ruiz J.R., Bettigole S.E., Glimcher L.H. (2017). Tumorigenic and immunosuppressive effects of endoplasmic reticulum stress in cancer. Cell.

[160] Mujcic H., Nagelkerke A., Rouschop K.M., Chung S., Chaudary N., Span P.N., Clarke B., Milosevic M., Sykes J., Hill R.P. (2013). Hypoxic activation of the PERK/eIF2α arm of the unfolded protein response promotes metastasis through induction of LAMP3. Clin. Cancer Res..

[161] Zhu H., Chen X., Chen B., Chen B., Song W., Sun D., Zhao Y. (2014). Activating transcription factor 4 promotes esophageal squamous cell carcinoma invasion and metastasis in mice and is associated with poor prognosis in human patients. PLoS ONE.

[162] Thevenot P.T., Sierra R.A., Raber P.L., Al-Khami A.A., Trillo-Tinoco J., Zarreii P., Ochoa A.C., Cui Y., Del Valle L., Rodriguez P.C. (2014). The stress-response sensor chop regulates the function and accumulation of myeloid-derived suppressor cells in tumors. Immunity.

[163] Condamine T., Kumar V., Ramachandran I.R., Youn J.I., Celis E., Finnberg N., El-Deiry W.S., Winograd R., Vonderheide R.H., English N.R. (2014). ER stress regulates myeloid-derived suppressor cell fate through trail-r–mediated apoptosis. J. Clin. Investig..

[164] Cubillos-Ruiz J.R., Silberman P.C., Rutkowski M.R., Chopra S., Perales-Puchalt A., Song M., Zhang S., Bettigole S.E., Gupta D., Holcomb K. (2015). ER stress sensor xbp1 controls anti-tumor immunity by disrupting dendritic cell homeostasis. Cell.

[165] Herber D.L., Cao W., Nefedova Y., Novitskiy S.V., Nagaraj S., Tyurin V.A., Corzo A., Cho H.I., Celis E., Lennox B. (2010). Lipid accumulation and dendritic cell dysfunction in cancer. Nat. Med..

[166] Hossain F., Al-Khami A.A., Wyczechowska D., Hernandez C., Zheng L., Reiss K., Del Valle L., Trillo-Tinoco J., Maj T., Zou W. (2015). Inhibition of fatty acid oxidation modulates immunosuppressive functions of myeloid-derived suppressor cells and enhances cancer therapies. Cancer Immunol. Res..

[167] Cao W., Ramakrishnan R., Tuyrin V.A., Veglia F., Condamine T., Amoscato A., Mohammadyani D., Johnson J.J., Zhang L.M., Klein-Seetharaman J. (2014). Oxidized lipids block antigen cross-presentation by dendritic cells in cancer. J. Immunol..

[168] Yan D., Wang H.W., Bowman R.L., Joyce J.A. (2016). Stat3 and stat6 signaling pathways synergize to promote cathepsin secretion from macrophages via ire1α activation. Cell Rep..

[169] Condamine T., Dominguez G.A., Youn J.I., Kossenkov A.V., Mony S., Alicea-Torres K., Tcyganov E., Hashimoto A., Nefedova Y., Lin C. (2016). Lectin-type oxidized LDL receptor-1 distinguishes population of human polymorphonuclear myeloid-derived suppressor cells in cancer patients. Sci. Immunol..

[170] Tang C.H.A., Ranatunga S., Kriss C.L., Cubitt C.L., Tao J., Pinilla-Ibarz J.A., Del Valle J.R., Hu C.C.A. (2014). Inhibition of ER stress–associated IRE-1/XBP-1 pathway reduces leukemic cell survival. J. Clin. Investig..

[171] Todd D.J., Lee A.H., Glimcher L.H. (2008). The endoplasmic reticulum stress response in immunity and autoimmunity. Nat. Rev. Immunol..

[172] Hetz C., Chevet E., Harding H.P. (2013). Targeting the unfolded protein response in disease. Nat. Rev. Drug Discov..

[173] Axten J.M., Medina J.S.R., Feng Y., Shu A., Romeril S.P., Grant S.W., Li W.H.H., Heerding D.A., Minthorn E., Mencken T. (2012). Discovery of 7-methyl-5-(1-{[3-(trifluoromethyl) phenyl] acetyl}-2, 3-dihydro-1 h-indol-5-yl)-7 h-pyrrolo [2, 3-d] pyrimidin-4-amine (GSK2606414), a potent and selective first-in-class inhibitor of protein kinase r (PKR)-like endoplasmic reticulum kinase (PERK). J Med. Chem..

[174] Atkins C., Liu Q., Minthorn E., Zhang S.Y., Figueroa D.J., Moss K., Stanley T.B., Sanders B., Goetz A., Gaul N. (2013). Characterization of a novel perk kinase inhibitor with antitumor and antiangiogenic activity. Cancer Res..

[175] Rouschop K.M., Dubois L.J., Keulers T.G., van den Beucken T., Lambin P., Bussink J., van der Kogel A.J., Koritzinsky M., Wouters B.G. (2013). PERK/eIF2α signaling protects therapy resistant hypoxic cells through induction of glutathione synthesis and protection against ROS. Proc. Natl. Acad. Sci. USA.

[176] Teng Y., Gao M., Wang J., Kong Q., Hua H., Luo T., Jiang Y. (2014). Inhibition of eIF2α dephosphorylation enhances TRAIL-induced apoptosis in hepatoma cells. Cell Death Dis..

[177] Hamamura K., Minami K., Tanjung N., Wan Q., Koizumi M., Matsuura N., Na S., Yokota H. (2014). Attenuation of malignant phenotypes of breast cancer cells through eIF2α-mediated downregulation of Rac1 signaling. Int. J. Oncol..

[178] Boyce M., Bryant K.F., Jousse C., Long K., Harding H.P., Scheuner D., Kaufman R.J., Ma D., Coen D.M., Ron D. (2005). A selective inhibitor of eIF2α dephosphorylation protects cells from ER stress. Science.

[179] Tsaytler P., Harding H.P., Ron D., Bertolotti A. (2011). Selective inhibition of a regulatory subunit of protein phosphatase 1 restores proteostasis. Science.

[180] Sidrauski C., McGeachy A.M., Ingolia N.T., Walter P. (2015). The small molecule isrib reverses the effects of eIF2α phosphorylation on translation and stress granule assembly. Elife.

[181] Cross B.C., Bond P.J., Sadowski P.G., Jha B.K., Zak J., Goodman J.M., Silverman R.H., Neubert T.A., Baxendale I.R., Ron D. (2012). The molecular basis for selective inhibition of unconventional mrna splicing by an ire1-binding small molecule. Proc. Natl. Acad. Sci. USA.

[182] Volkmann K., Lucas J.L., Vuga D., Wang X., Brumm D., Stiles C., Kriebel D., Der-Sarkissian A., Krishnan K., Schweitzer C. (2011). Potent and selective inhibitors of the inositol-requiring enzyme 1 endoribonuclease. J. Biol. Chem..

[183] Suh D.H., Kim M.K., Kim H.S., Chung H.H., Song Y.S. (2012). Unfolded protein response to autophagy as a promising druggable target for anticancer therapy. Ann. N. Y. Acad. Sci..

[184] Ming J., Ruan S., Wang M., Ye D., Fan N., Meng Q., Tian B., Huang T. (2015). A novel chemical, STF-083010, reverses tamoxifen-related drug resistance in breast cancer by inhibiting IRE1/XBP1. Oncotarget.

[185] Jiang D., Tam A.B., Alagappan M., Hay M.P., Gupta A., Kozak M.M., Solow-Cordero D.E., Lum P.Y., Denko N.C., Giaccia A.J. (2016). Acridine derivatives as inhibitors of the ire1α–xbp1 pathway are cytotoxic to human multiple myeloma. Mol. Cancer Ther..

[186] Sanches M., Duffy N.M., Talukdar M., Thevakumaran N., Chiovitti D., Canny M.D., Lee K., Kurinov I., Uehling D., Al-Awar R. (2014). Structure and mechanism of action of the hydroxy–aryl–aldehyde class of ire1 endoribonuclease inhibitors. Nat. Commun..

[187] Vogelzangs N., Duivis H.E., Beekman A.T., Kluft C., Neuteboom J., Hoogendijk W., Smit J.H., de Jonge P., Penninx B.W. (2012). Association of depressive disorders, depression characteristics and antidepressant medication with inflammation. Transl. Psychiatry.

[188] Wang M., Law M.E., Castellano R.K., Law B.K. (2018). The unfolded protein response as a target for anticancer therapeutics. Crit. Rev. Oncol. Hematol..

[189] Wang L., Perera B.G.K., Hari S.B., Bhhatarai B., Backes B.J., Seeliger M.A., Schürer S.C., Oakes S.A., Papa F.R., Maly D.J. (2012). Divergent allosteric control of the ire1α endoribonuclease using kinase inhibitors. Nat. Chem. Biol..

[190] Wang F.M., Galson D.L., Roodman G.D., Ouyang H. (2011). Resveratrol triggers the pro-apoptotic endoplasmic reticulum stress response and represses pro-survival XBP1 signaling in human multiple myeloma cells. Exp. Hematol..

[191] Rojas C., Pan-Castillo B., Valls C., Pujadas G., Garcia-Vallve S., Arola L., Mulero M. (2014). Resveratrol enhances palmitate-induced ER stress and apoptosis in cancer cells. PLoS ONE.

[192] Hetz C. (2012). The unfolded protein response: Controlling cell fate decisions under ER stress and beyond. Nat. Rev. Mol. Cell Biol..

[193] Gallagher C.M., Walter P. (2016). Ceapins inhibit atf6α signaling by selectively preventing transport of ATF6α to the Golgi apparatus during ER stress. Elife.

[194] Brodsky J.L., Wojcikiewicz R.J. (2009). Substrate-specific mediators of ER associated degradation (ERAD). Curr. Opin. Cell Biol..

[195] Hampton R.Y. (2002). ER-associated degradation in protein quality control and cellular regulation. Curr. Opin. Cell Biol..

[196] Hershko A., Ciechanover A. (1998). The ubiquitin system. Annu. Rev. Biochem..

[197] Suraweera A., Münch C., Hanssum A., Bertolotti A. (2012). Failure of amino acid homeostasis causes cell death following proteasome inhibition. Mol. Cell.

[198] Kisselev A.F., van der Linden W.A., Overkleeft H.S. (2012). Proteasome inhibitors: An expanding army attacking a unique target. Chem. Biol..

[199] Begg A.C., Stewart F.A., Vens C. (2011). Strategies to improve radiotherapy with targeted drugs. Nat. Rev. Cancer.

[200] Manasanch E.E., Orlowski R.Z. (2017). Proteasome inhibitors in cancer therapy. Nat. Rev. Clin. Oncol..

[201] Fribley A., Zeng Q., Wang C.Y. (2004). Proteasome inhibitor PS-341 induces apoptosis through induction of endoplasmic reticulum stress-reactive oxygen species in head and neck squamous cell carcinoma cells. Mol. Cell. Biol..

[202] Ciombor K.K., Feng Y., Su Y., Horton L., Short S.P., Kauh J.S.W., Staley C., Mulcahy M., Powell M., Amiri K.I. (2014). Phase ii trial of bortezomib plus doxorubicin in hepatocellular carcinoma (e6202): A trial of the eastern cooperative oncology group. Investig. New Drugs.

[203] Nawrocki S.T., Carew J.S., Dunner K., Boise L.H., Chiao P.J., Huang P., Abbruzzese J.L., McConkey D.J. (2005). Bortezomib inhibits PKR-like endoplasmic reticulum (ER) kinase and induces apoptosis via ER stress in human pancreatic cancer cells. Cancer Res..

[204] Roccaro A.M., Hideshima T., Raje N., Kumar S., Ishitsuka K., Yasui H., Shiraishi N., Ribatti D., Nico B., Vacca A. (2006). Bortezomib mediates antiangiogenesis in multiple myeloma via direct and indirect effects on endothelial cells. Cancer Res..

[205] Sunwoo J.B., Chen Z., Dong G., Yeh N., Bancroft C.C., Sausville E., Adams J., Elliott P., Van Waes C. (2001). Novel proteasome inhibitor PS-341 inhibits activation of nuclear factor-κB, cell survival, tumor growth, and angiogenesis in squamous cell carcinoma. Clin. Cancer Res..

[206] Politou M., Naresh K., Terpos E., Crawley D., Lampert I., Apperley J.F., Rahemtulla A. (2005). Anti-angiogenic effect of bortezomib in patients with multiple myeloma. Acta Haematol..

[207] Richardson P.G., Xie W., Jagannath S., Jakubowiak A., Lonial S., Raje N.S., Alsina M., Ghobrial I.M., Schlossman R.L., Munshi N.C. (2014). A phase 2 trial of lenalidomide, bortezomib, and dexamethasone in patients with relapsed and relapsed/refractory myeloma. Blood.

[208] Roy S.S., Kirma N.B., Santhamma B., Tekmal R.R., Agyin J.K. (2014). Effects of a novel proteasome inhibitor bu-32 on multiple myeloma cells. Cancer Chemother. Pharmacol..

[209] Agyin J.K., Santhamma B., Nair H.B., Roy S.S., Tekmal R.R. (2009). Bu-32: A novel proteasome inhibitor for breast cancer. Breast Cancer Res..

[210] Ping Dou Q., Zonder J.A. (2014). Overview of proteasome inhibitor-based anti-cancer therapies: Perspective on bortezomib and second generation proteasome inhibitors versus future generation inhibitors of ubiquitin-proteasome system. Curr. Cancer Drug Targets.

[211] Garcia-Gomez A., Quwaider D., Canavese M., Ocio E.M., Tian Z., Blanco J.F., Berger A.J., Ortiz-de-Solorzano C., Hernández-Iglesias T., Martens A.C. (2014). Preclinical activity of the oral proteasome inhibitor mln9708 in myeloma bone disease. Clin. Cancer Res..

[212] Crawford L.J., Walker B., Irvine A.E. (2011). Proteasome inhibitors in cancer therapy. J. Cell Commun. Signal..

[213] Goldberg A.L. (2012). Development of Proteasome Inhibitors as Research Tools and Cancer Drugs.

[214] Moreau P., Richardson P.G., Cavo M., Orlowski R.Z., San Miguel J.F., Palumbo A., Harousseau J.L. (2012). Proteasome inhibitors in multiple myeloma: 10 years later. Blood.

[215] Obrist F., Manic G., Kroemer G., Vitale I., Galluzzi L. (2015). Trial watch: Proteasomal inhibitors for anticancer therapy. Mol. Cell. Oncol..

[216] Badin F.B., Chiang A.C., Fisher W.B., Orlov S., Harper H.D., Eskander E., Harb W.A., Kio E., Gopalan P.K., Haggstrom D.E. (2016). Carfilzomib (CFZ), carboplatin and etoposide for previously untreated extensive-stage small cell lung cancer (ES-SCLC): Phase 1b results from a phase 1b/2 study. J. Clin. Oncol..

[217] Schönthal A.H. (2012). Endoplasmic reticulum stress: Its role in disease and novel prospects for therapy. Scientifica.

[218] Kazi A., Daniel K.G., Smith D.M., Kumar N.B., Dou Q.P. (2003). Inhibition of the proteasome activity, a novel mechanism associated with the tumor cell apoptosis-inducing ability of genistein. Biochem. Pharmacol..

[219] Chen D., Chen M.S., Cui Q.C., Yang H., Dou Q.P. (2007). Structure-proteasome-inhibitory activity relationships of dietary flavonoids in human cancer cells. Front. Biosci..

[220] Chen D., Landis-Piwowar K.R., Chen M.S., Dou Q.P. (2007). Inhibition of proteasome activity by the dietary flavonoid apigenin is associated with growth inhibition in cultured breast cancer cells and xenografts. Breast Cancer Res..

[221] Chen D., Daniel K.G., Chen M.S., Kuhn D.J., Landis-Piwowar K.R., Dou Q.P. (2005). Dietary flavonoids as proteasome inhibitors and apoptosis inducers in human leukemia cells. Biochem. Pharmacol..

[222] Jana N.R., Dikshit P., Goswami A., Nukina N. (2004). Inhibition of proteasomal function by curcumin induces apoptosis through mitochondrial pathway. J. Biol. Chem..

[223] Nam S., Smith D.M., Dou Q.P. (2001). Tannic acid potently inhibits tumor cell proteasome activity, increases p27 and Bax expression, and induces g1 arrest and apoptosis. Cancer Epidemiol. Prev. Biomark..

[224] Saiko P., Steinmann M.T., Schuster H., Graser G., Bressler S., Giessrigl B., Lackner A., Grusch M., Krupitza G., Bago-Horvath Z. (2015). Epigallocatechin gallate, ellagic acid, and rosmarinic acid perturb dNTP pools and inhibit de novo DNA synthesis and proliferation of human HL-60 promyelocytic leukemia cells: Synergism with arabinofuranosylcytosine. Phytomedicine.

[225] Wang P., Henning S.M., Heber D., Vadgama J.V. (2015). Sensitization to docetaxel in prostate cancer cells by green tea and quercetin. J. Nutr. Biochem..

[226] Ho Zhi Guang M., Kavanagh E.L., Dunne L.P., Dowling P., Zhang L., Lindsay S., Bazou D., Goh C.Y., Hanley C., Bianchi G. (2019). Targeting proteotoxic stress in cancer: A review of the role that protein quality control pathways play in oncogenesis. Cancers.

[227] Vogl D.T., Stadtmauer E.A., Tan K.S., Heitjan D.F., Davis L.E., Pontiggia L., Rangwala R., Piao S., Chang Y.C., Scott E.C. (2014). Combined autophagy and proteasome inhibition: A phase 1 trial of hydroxychloroquine and bortezomib in patients with relapsed/refractory myeloma. Autophagy.

[228] Jakubowiak A., Offidani M., Pégourie B., De La Rubia J., Garderet L., Laribi K., Bosi A., Marasca R., Laubach J., Mohrbacher A. (2016). Randomized phase 2 study: Elotuzumab plus bortezomib/dexamethasone vs bortezomib/dexamethasone for relapsed/refractory mm. Blood.

[229] Palumbo A., Chanan-Khan A., Weisel K., Nooka A.K., Masszi T., Beksac M., Spicka I., Hungria V., Munder M., Mateos M.V. (2016). Daratumumab, bortezomib, and dexamethasone for multiple myeloma. N. Engl. J. Med..

[230] San-Miguel J.F., Hungria V.T., Yoon S.S., Beksac M., Dimopoulos M.A., Elghandour A., Jedrzejczak W.W., Günther A., Nakorn T.N., Siritanaratkul N. (2014). Panobinostat plus bortezomib and dexamethasone versus placebo plus bortezomib and dexamethasone in patients with relapsed or relapsed and refractory multiple myeloma: A multicentre, randomised, double-blind phase 3 trial. Lancet Oncol..

[231] San-Miguel J.F., Hungria V.T., Yoon S.S., Beksac M., Dimopoulos M.A., Elghandour A., Jedrzejczak W.W., Günther A., Nakorn T.N., Siritanaratkul N. (2016). Overall survival of patients with relapsed multiple myeloma treated with panobinostat or placebo plus bortezomib and dexamethasone (the panorama 1 trial): A randomised, placebo-controlled, phase 3 trial. Lancet Haematol..

[232] Moreau P., Chanan-Khan A., Roberts A.W., Agarwal A.B., Facon T., Kumar S., Touzeau C., Punnoose E.A., Cordero J., Munasinghe W. (2017). Promising efficacy and acceptable safety of venetoclax plus bortezomib and dexamethasone in relapsed/refractory mm. Blood.

[233] Santo L., Hideshima T., Kung A.L., Tseng J.C., Tamang D., Yang M., Jarpe M., van Duzer J.H., Mazitschek R., Ogier W.C. (2012). Preclinical activity, pharmacodynamic, and pharmacokinetic properties of a selective hdac6 inhibitor, acy-1215, in combination with bortezomib in multiple myeloma. Blood.

[234] Mishima Y., Santo L., Eda H., Cirstea D., Nemani N., Yee A.J., O’Donnell E., Selig M.K., Quayle S.N., Arastu-Kapur S. (2015). Ricolinostat (acy-1215) induced inhibition of aggresome formation accelerates carfilzomib-induced multiple myeloma cell death. Br. J. Haematol..

[235] Petrocca F., Altschuler G., Tan S.M., Mendillo M.L., Yan H., Jerry D.J., Kung A.L., Hide W., Ince T.A., Lieberman J. (2013). A genome-wide sirna screen identifies proteasome addiction as a vulnerability of basal-like triple-negative breast cancer cells. Cancer Cell.

[236] Chen Y.J., Yeh M.H., Yu M.C., Wei Y.L., Chen W.S., Chen J.Y., Shih C.Y., Tu C.Y., Chen C.H., Hsia T.C. (2013). Lapatinib–induced NF-kappaB activation sensitizes triple-negative breast cancer cells to proteasome inhibitors. Breast Cancer Res..

[237] Richardson P.G., Sonneveld P., Schuster M.W., Stadtmauer E.A., Facon T., Harousseau J.L., Ben-Yehuda D., Lonial S., Goldschmidt H., Reece D. (2009). Reversibility of symptomatic peripheral neuropathy with bortezomib in the phase iii apex trial in relapsed multiple myeloma: Impact of a dose-modification guideline. Br. J. Haematol..

[238] Richardson P.G., Sonneveld P., Schuster M.W., Irwin D., Stadtmauer E.A., Facon T., Harousseau J.L., Ben-Yehuda D., Lonial S., Goldschmidt H. (2005). Bortezomib or high-dose dexamethasone for relapsed multiple myeloma. N. Engl. J. Med..

[239] Lonial S., Richardson P.G., San Miguel J., Sonneveld P., Schuster M.W., Bladé J., Cavenagh J., Rajkumar S.V., Jakubowiak A.J., Esseltine D.L. (2008). Characterisation of haematological profiles and low risk of thromboembolic events with bortezomib in patients with relapsed multiple myeloma. Br. J. Haematol..

[240] Chanan-Khan A., Sonneveld P., Schuster M.W., Stadtmauer E.A., Facon T., Harousseau J.L., Ben-Yehuda D., Lonial S., Goldschmidt H., Reece D. (2008). Analysis of herpes zoster events among bortezomib-treated patients in the phase iii apex study. J. Clin. Oncol..

[241] Grandin E.W., Ky B., Cornell R.F., Carver J., Lenihan D.J. (2015). Patterns of cardiac toxicity associated with irreversible proteasome inhibition in the treatment of multiple myeloma. J. Card. Fail..

[242] Danhof S., Schreder M., Rasche L., Strifler S., Einsele H., Knop S. (2016). ‘Real-life’experience of preapproval carfilzomib-based therapy in myeloma–analysis of cardiac toxicity and predisposing factors. Eur. J. Haematol..

[243] Dimopoulos M.A., Roussou M., Gavriatopoulou M., Psimenou E., Ziogas D., Eleutherakis-Papaiakovou E., Fotiou D., Migkou M., Kanellias N., Panagiotidis I. (2017). Cardiac and renal complications of carfilzomib in patients with multiple myeloma. Blood Adv..

[244] Brem G.J., Mylonas I., Brüning A. (2013). Eeyarestatin causes cervical cancer cell sensitization to bortezomib treatment by augmenting ER stress and chop expression. Gynecol. Oncol..

[245] Chou T.F., Brown S.J., Minond D., Nordin B.E., Li K., Jones A.C., Chase P., Porubsky P.R., Stoltz B.M., Schoenen F.J. (2011). Reversible inhibitor of p97, dbeq, impairs both ubiquitin-dependent and autophagic protein clearance pathways. Proc. Natl. Acad. Sci. USA.

[246] Chou T.F., Li K., Frankowski K.J., Schoenen F.J., Deshaies R.J. (2013). Structure–activity relationship study reveals ML240 and ML241 as potent and selective inhibitors of p97 ATPase. ChemMedChem.

[247] Polucci P., Magnaghi P., Angiolini M., Asa D., Avanzi N., Badari A., Bertrand J., Casale E., Cauteruccio S., Cirla A. (2013). Alkylsulfanyl-1, 2, 4-triazoles, a new class of allosteric valosine containing protein inhibitors. Synthesis and structure–activity relationships. J. Med. Chem..

[248] Valle C.W., Min T., Bodas M., Mazur S., Begum S., Tang D., Vij N. (2011). Critical role of VCP/p97 in the pathogenesis and progression of non-small cell lung carcinoma. PLoS ONE.

[249] Vekaria P.H., Home T., Weir S., Schoenen F.J., Rao R. (2016). Targeting p97 to disrupt protein homeostasis in cancer. Front. Oncol..

[250] Chen O.I., Bobak Y.P., Stasyk O.V., Kunz-Schughart L.A. (2018). A complex scenario and underestimated challenge: The tumor microenvironment, ER stress, and cancer treatment. Curr. Med. Chem..

[251] Wang Q., Li L., Ye Y. (2008). Inhibition of p97-dependent protein degradation by eeyarestatin I. J. Biol. Chem..

[252] Wang Q., Mora-Jensen H., Weniger M.A., Perez-Galan P., Wolford C., Hai T., Ron D., Chen W., Trenkle W., Wiestner A. (2009). ERAD inhibitors integrate ER stress with an epigenetic mechanism to activate bh3-only protein NOXA in cancer cells. Proc. Natl. Acad. Sci. USA.

[253] Anderson D.J., Le Moigne R., Djakovic S., Kumar B., Rice J., Wong S., Wang J., Yao B., Valle E., von Soly S.K. (2015). Targeting the AAA ATPase p97 as an approach to treat cancer through disruption of protein homeostasis. Cancer Cell.

[254] Zhou H.J., Wang J., Yao B., Wong S., Djakovic S., Kumar B., Rice J., Valle E., Soriano F., Menon M.K. (2015). Discovery of a first-in-class, potent, selective, and orally bioavailable inhibitor of the p97 AAA ATPase (CB-5083). J. Med. Chem..

[255] Le Moigne R., Aftab B.T., Djakovic S., Dhimolea E., Valle E., Murnane M., King E.M., Soriano F., Menon M.K., Wu Z.Y. (2017). The p97 inhibitor CB-5083 is a unique disrupter of protein homeostasis in models of multiple myeloma. Mol. Cancer Ther..

[256] Martin S., Lamb H., Brady C., Lefkove B., Bonner M., Thompson P., Lovat P., Arbiser J., Hawkins A., Redfern C. (2013). Inducing apoptosis of cancer cells using small-molecule plant compounds that bind to GRP78. Br. J. Cancer.

[257] Paton A.W., Beddoe T., Thorpe C.M., Whisstock J.C., Wilce M.C., Rossjohn J., Talbot U.M., Paton J.C. (2006). AB_5_ subtilase cytotoxin inactivates the endoplasmic reticulum chaperone BiP. Nature.

[258] Backer J.M., Krivoshein A.V., Hamby C.V., Pizzonia J., Gilbert K.S., Ray Y.S., Brand H., Paton A.W., Paton J.C., Backer M.V. (2009). Chaperone-targeting cytotoxin and endoplasmic reticulum stress-inducing drug synergize to kill cancer cells. Neoplasia.

[259] Firczuk M., Gabrysiak M., Barankiewicz J., Domagala A., Nowis D., Kujawa M., Jankowska-Steifer E., Wachowska M., Glodkowska-Mrowka E., Korsak B. (2013). Grp78-targeting subtilase cytotoxin sensitizes cancer cells to photodynamic therapy. Cell Death Dis..

[260] Cerezo M., Lehraiki A., Millet A., Rouaud F., Plaisant M., Jaune E., Botton T., Ronco C., Abbe P., Amdouni H. (2016). Compounds triggering ER stress exert anti-melanoma effects and overcome BRAF inhibitor resistance. Cancer Cell.

[261] Ermakova S.P., Kang B.S., Choi B.Y., Choi H.S., Schuster T.F., Ma W.Y., Bode A.M., Dong Z. (2006). (−)−epigallocatechin gallate overcomes resistance to etoposide-induced cell death by targeting the molecular chaperone glucose-regulated protein 78. Cancer Res..

[262] Matsuo J., Tsukumo Y., Sakurai J., Tsukahara S., Park H.R., Shin-ya K., Watanabe T., Tsuruo T., Tomida A. (2009). Preventing the unfolded protein response via aberrant activation of 4E-binding protein 1 by versipelostatin. Cancer Sci..

[263] Cook K.L., Clarke R. (2015). Role of GRP78 in promoting therapeutic-resistant breast cancer. Future Med. Chem..

[264] Kitao Y., Ozawa K., Miyazaki M., Tamatani M., Kobayashi T., Yanagi H., Okabe M., Ikawa M., Yamashima T., Stern D.M. (2001). Expression of the endoplasmic reticulum molecular chaperone (ORP150) rescues hippocampal neurons from glutamate toxicity. J. Clin. Investig..

[265] Wang Z.S., Lu F.E., Xu L.J., Dong H. (2010). Berberine reduces endoplasmic reticulum stress and improves insulin signal transduction in Hep G2 cells. Acta Pharmacol. Sin..

[266] Lawson B., Brewer J.W., Hendershot L.M. (1998). Geldanamycin, an hsp90/GRP94-binding drug, induces increased transcription of endoplasmic reticulum (ER) chaperones via the ER stress pathway. J. Cell. Physiol..

[267] Jones D.T., Addison E., North J.M., Lowdell M.W., Hoffbrand A.V., Mehta A.B., Ganeshaguru K., Folarin N.I., Wickremasinghe R.G. (2004). Geldanamycin and herbimycin a induce apoptotic killing of b chronic lymphocytic leukemia cells and augment the cells’ sensitivity to cytotoxic drugs. Blood.

[268] Booth L., Roberts J.L., Cruickshanks N., Conley A., Durrant D.E., Das A., Fisher P.B., Kukreja R.C., Grant S., Poklepovic A. (2014). Phosphodiesterase 5 inhibitors enhance chemotherapy killing in gastrointestinal/genitourinary cancer cells. Mol. Pharmacol..

[269] Booth L., Roberts J.L., Cruickshanks N., Grant S., Poklepovic A., Dent P. (2014). Regulation of osu-03012 toxicity by ER stress proteins and ER stress–inducing drugs. Mol. Cancer Ther..

[270] Jhaveri K., Taldone T., Modi S., Chiosis G. (2012). Advances in the clinical development of heat shock protein 90 (Hsp90) inhibitors in cancers. Biochim. Biophys. Acta BBA Mol. Cell Res..

[271] Neckers L., Workman P. (2012). Hsp90 molecular chaperone inhibitors: Are we there yet?. Clin. Cancer Res..

[272] Davenport E.L., Moore H.E., Dunlop A.S., Sharp S.Y., Workman P., Morgan G.J., Davies F.E. (2007). Heat shock protein inhibition is associated with activation of the unfolded protein response pathway in myeloma plasma cells. Blood.

[273] De Raedt T., Walton Z., Yecies J.L., Li D., Chen Y., Malone C.F., Maertens O., Jeong S.M., Bronson R.T., Lebleu V. (2011). Exploiting cancer cell vulnerabilities to develop a combination therapy for ras-driven tumors. Cancer Cell.

[274] Duerfeldt A.S., Peterson L.B., Maynard J.C., Ng C.L., Eletto D., Ostrovsky O., Shinogle H.E., Moore D.S., Argon Y., Nicchitta C.V. (2012). Development of a Grp94 inhibitor. J. Am. Chem. Soc..

[275] Duerfeldt A.S., Brandt G.E., Blagg B.S. (2009). Design, synthesis, and biological evaluation of conformationally constrained cis-amide hsp90 inhibitors. Org. Lett..

[276] Goplen D., Wang J., Enger P.Ø., Tysnes B.B., Terzis A., Laerum O.D., Bjerkvig R. (2006). Protein disulfide isomerase expression is related to the invasive properties of malignant glioma. Cancer Res..

[277] Lovat P.E., Corazzari M., Armstrong J.L., Martin S., Pagliarini V., Hill D., Brown A.M., Piacentini M., Birch-Machin M.A., Redfern C.P. (2008). Increasing melanoma cell death using inhibitors of protein disulfide isomerases to abrogate survival responses to endoplasmic reticulum stress. Cancer Res..

[278] Garg A.D., Nowis D., Golab J., Vandenabeele P., Krysko D.V., Agostinis P. (2010). Immunogenic cell death, damps and anticancer therapeutics: An emerging amalgamation. Biochim. Biophys. Acta BBA Rev. Cancer.

[279] Garg A.D., Martin S., Golab J., Agostinis P. (2014). Danger signalling during cancer cell death: Origins, plasticity and regulation. Cell Death Differ..

[280] Krysko D.V., Garg A.D., Kaczmarek A., Krysko O., Agostinis P., Vandenabeele P. (2012). Immunogenic cell death and damps in cancer therapy. Nat. Rev. Cancer.

[281] Zitvogel L., Kepp O., Senovilla L., Menger L., Chaput N., Kroemer G. (2010). Immunogenic tumor cell death for optimal anticancer therapy: The calreticulin exposure pathway. Clin. Cancer Res..

[282] Van Vliet A., Martin S., Garg A., Agostinis P. (2015). The perks of damage-associated molecular patterns mediating cancer immunogenicity: From sensor to the plasma membrane and beyond. Semin. Cancer Biol..

[283] Garg A.D., Krysko D.V., Verfaillie T., Kaczmarek A., Ferreira G.B., Marysael T., Rubio N., Firczuk M., Mathieu C., Roebroek A.J. (2012). A novel pathway combining calreticulin exposure and ATP secretion in immunogenic cancer cell death. EMBO J..

[284] Panaretakis T., Kepp O., Brockmeier U., Tesniere A., Bjorklund A.C., Chapman D.C., Durchschlag M., Joza N., Pierron G., Van Endert P. (2009). Mechanisms of pre-apoptotic calreticulin exposure in immunogenic cell death. EMBO J..

[285] Garg A.D., Dudek A.M., Ferreira G.B., Verfaillie T., Vandenabeele P., Krysko D.V., Mathieu C., Agostinis P. (2013). Ros-induced autophagy in cancer cells assists in evasion from determinants of immunogenic cell death. Autophagy.

[286] Garg A.D., Dudek A.M., Agostinis P. (2013). Calreticulin surface exposure is abrogated in cells lacking, chaperone-mediated autophagy-essential gene, LAMP2A. Cell Death Dis..

[287] Michaud M., Martins I., Sukkurwala A.Q., Adjemian S., Ma Y., Pellegatti P., Shen S., Kepp O., Scoazec M., Mignot G. (2011). Autophagy-dependent anticancer immune responses induced by chemotherapeutic agents in mice. Science.

[288] Garg A.D., Maes H., van Vliet A.R., Agostinis P. (2015). Targeting the hallmarks of cancer with therapy-induced endoplasmic reticulum (ER) stress. Mol. Cell. Oncol..

[289] Fucikova J., Becht E., Iribarren K., Goc J., Remark R., Damotte D., Alifano M., Devi P., Biton J., Germain C. (2016). Calreticulin expression in human non–small cell lung cancers correlates with increased accumulation of antitumor immune cells and favorable prognosis. Cancer Res..

[290] Shoulders M.D., Ryno L.M., Genereux J.C., Moresco J.J., Tu P.G., Wu C., Yates J.R., Su A.I., Kelly J.W., Wiseman R.L. (2013). Stress-independent activation of XBP1s and/or ATF6 reveals three functionally diverse ER proteostasis environments. Cell Rep..

[291] Kepp O., Menger L., Vacchelli E., Locher C., Adjemian S., Yamazaki T., Martins I., Sukkurwala A.Q., Michaud M., Senovilla L. (2013). Crosstalk between ER stress and immunogenic cell death. Cytokine Growth Factor Rev..

[292] Wang S., Kaufman R.J. (2012). The impact of the unfolded protein response on human disease. J. Cell Biol..

[293] Hetz C., Chevet E., Oakes S.A. (2015). Proteostasis control by the unfolded protein response. Nat. Cell Biol..

[294] Field-Smith A., Morgan G.J., Davies F.E. (2006). Bortezomib (velcade™) in the treatment of multiple myeloma. Ther. Clin. Risk Manag..

[295] Obeng E.A., Carlson L.M., Gutman D.M., Harrington W.J., Lee K.P., Boise L.H. (2006). Proteasome inhibitors induce a terminal unfolded protein response in multiple myeloma cells. Blood.

